# Gasdermin D inhibition confers antineutrophil-mediated cardioprotection in acute myocardial infarction

**DOI:** 10.1172/JCI151268

**Published:** 2022-01-04

**Authors:** Kai Jiang, Zizhuo Tu, Kun Chen, Yue Xu, Feng Chen, Sheng Xu, Tingting Shi, Jie Qian, Lan Shen, John Hwa, Dandan Wang, Yaozu Xiang

**Affiliations:** 1 Shanghai East Hospital, Key Laboratory of Arrhythmias of the Ministry of Education of China, School of Life Sciences and Technology, Tongji University, Shanghai, China.; 2 Shanghai Tenth People’s Hospital, Tongji University School of Medicine, Shanghai, China.; 3 Department of Cardiology, Clinical Research Unit, Shanghai Chest Hospital, Shanghai Jiao Tong University, Shanghai, China.; 4 Section of Cardiovascular Medicine, Department of Internal Medicine, Yale Cardiovascular Research Center, Yale University School of Medicine, New Haven, Connecticut, USA.

**Keywords:** Cardiology, Neutrophils

## Abstract

Acute myocardial infarction (AMI) induces blood leukocytosis, which correlates inversely with patient survival. The molecular mechanisms leading to leukocytosis in the infarcted heart remain poorly understood. Using an AMI mouse model, we identified gasdermin D (GSDMD) in activated leukocytes early in AMI. We demonstrated that GSDMD is required for enhanced early mobilization of neutrophils to the infarcted heart. Loss of GSDMD resulted in attenuated IL-1**β** release from neutrophils and subsequent decreased neutrophils and monocytes in the infarcted heart. Knockout of GSDMD in mice significantly reduced infarct size, improved cardiac function, and increased post-AMI survival. Through a series of bone marrow transplantation studies and leukocyte depletion experiments, we further clarified that excessive bone marrow–derived and GSDMD-dependent early neutrophil production and mobilization (24 hours after AMI) contributed to the detrimental immunopathology after AMI. Pharmacological inhibition of GSDMD also conferred cardioprotection after AMI through a reduction in scar size and enhancement of heart function. Our study provides mechanistic insights into molecular regulation of neutrophil generation and mobilization after AMI, and supports GSDMD as a new target for improved ventricular remodeling and reduced heart failure after AMI.

## Graphical abstract

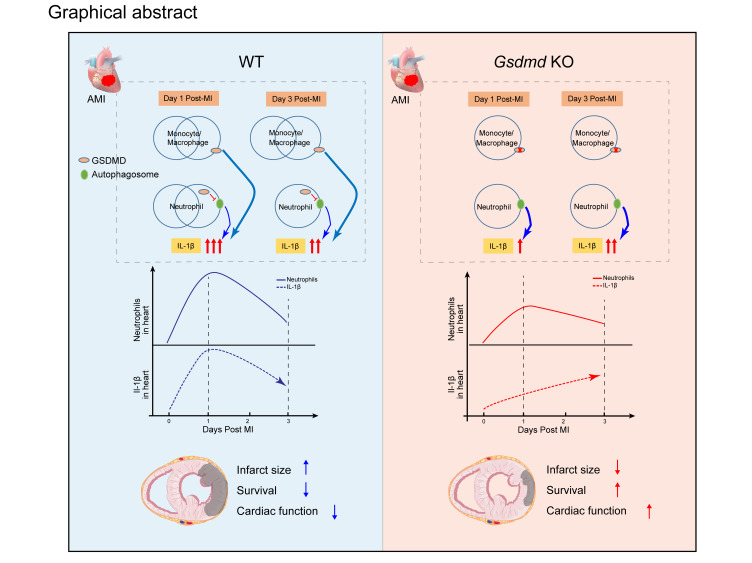

## Introduction

Acute myocardial infarction (AMI) is a leading cause of death worldwide. Although reperfusion is successful in reducing infarct size and improving overall prognosis, AMI remains a major cause of heart failure and increased morbidity and mortality (1). In the past 2 decades, an increase in programmed cardiomyocyte cell death has been recognized in AMI and ischemia–reperfusion (I/R) injury (1, 2). Sudden massive loss of cardiomyocytes after AMI exceeds the limited regenerative capacity of the myocardium (3). Cytokines released from necrotic cells can activate innate immune pathways, triggering an intense inflammatory response (4). Dysregulation of the inflammatory response may cause adverse remodeling (fibrosis and scar formation) in patients with AMI, contributing to postinfarction heart failure (5). Therapeutic attempts to suppress inflammation during AMI can lead to impaired cardiac repair and increased risk of cardiac rupture (6). More recent strategies aimed at selectively blocking key inflammatory factors rather than globally suppressing the response have shown some promising results (7).

Accumulating evidence has underscored a central role of inflammasomes in AMI (8). The most widely characterized inflammasome sensor in the heart is the NACHT, LRR, and PYD domain–containing protein 3 (NLRP3), which is activated in response to cell debris during AMI (8). Activation of the NLRP3 inflammasome triggers myocardial damage through promotion of inflammatory cell death via pyroptosis and through release of interleukin 1β (IL-1β) (8). In contrast, downregulation or inhibition of inflammasome components, including NLRP3 (9), the adapter apoptosis-associated speck-like (ASC) protein, and caspase-1, may reduce infarct size (8, 10). However, inhibition of IL‑1β activity appears not to reduce infarct size (11). The pyroptotic substrate is the pore-forming protein gasdermin D (GSDMD) (12–14), which is widely expressed in different subsets of leukocytes (15). Cardiac neutrophil and monocyte/macrophage numbers expand rapidly in the days following AMI (16). Recent findings suggest that GSDMD plays a distinct role in neutrophils during inflammasome activation, which differs from its role in macrophages (17, 18). The regulatory role of GSDMD in response to AMI is unknown.

We now demonstrate that GSDMD is activated early in AMI and plays a critical role in increased production and mobilization of neutrophils. Both genetic deletion and pharmacological inhibition of GSDMD attenuated myocardial injury, reduced infarct size, and improved cardiac function and survival. We further demonstrated that GSDMD deficiency reduced acute cardiac cell death and IL-1β production independently of NLRP3 inflammasome activation. Consequently, reduced leukocyte numbers in the blood (and infarct) decreased inflammation and diminished post-AMI heart failure. Our work thus identifies GSDMD-dependent, bone marrow–derived neutrophil generation and mobilization as an important contributing factor to cardiac immunopathology after AMI, and provides mechanistic insights into the modulation of inflammatory responses during AMI, involving pyroptosis-dependent and pyroptosis-independent regulatory networks.

## Results

### GSDMD is activated in the early phase of AMI.

**Figure 1:**
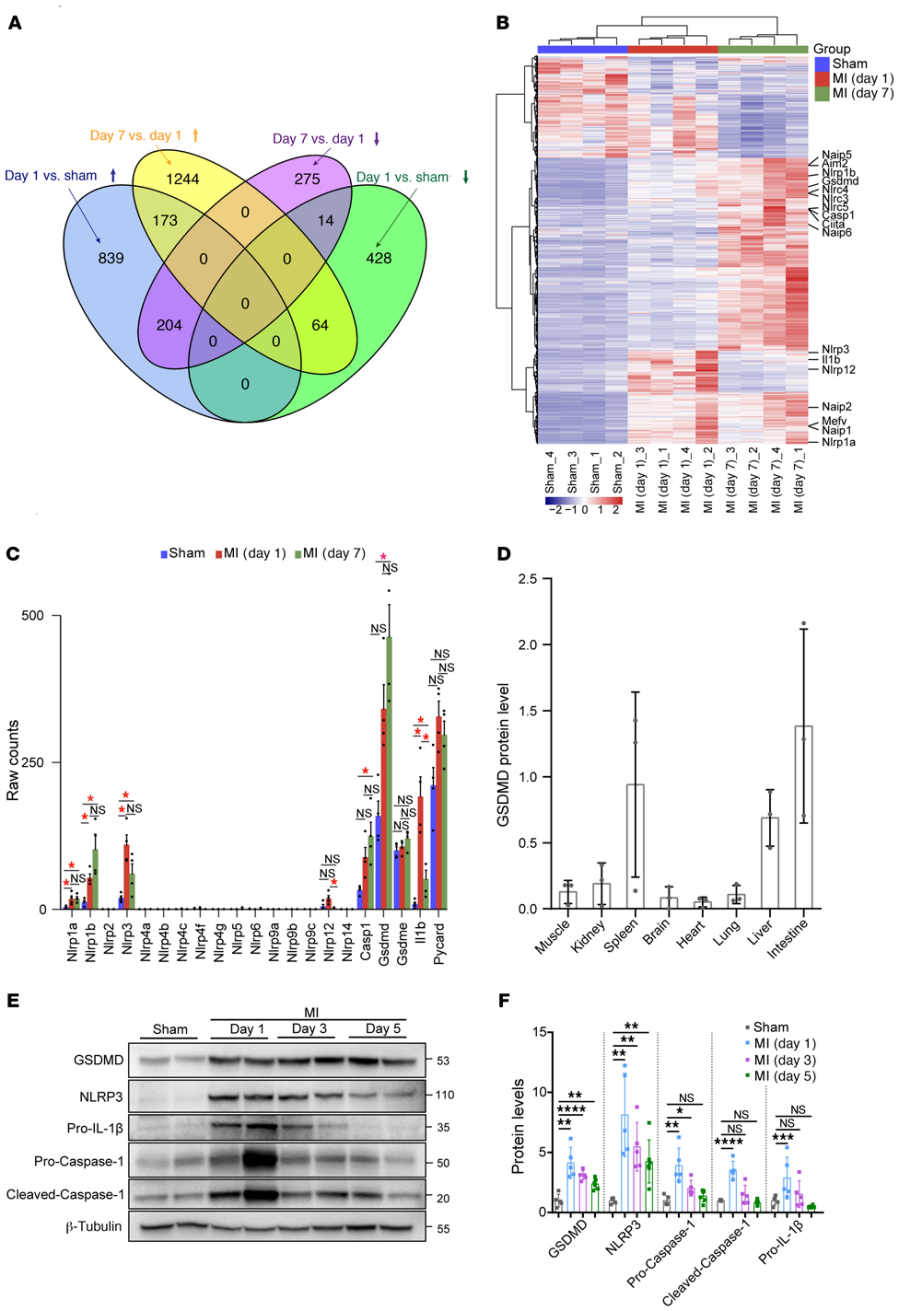
GSDMD is activated in the early phase of AMI. (**A**) Venn diagram revealing the intersection of differentially expressed genes created from the comparisons of day 1 AMI vs. sham and day 7 AMI vs. day 1 AMI. (**B**) Each row in the heatmap represents a specific gene that had significantly different expression levels in comparisons between any two groups, the expression of which was normalized across the column, with high expression shown in red and low in blue. (**C**) Bar plot showing the trends of gene expression across sham, day 1 AMI, and day 7 AMI. *Indicates a statistically significant difference, with fold change (FC) ≥ 2 and false discovery rate (FDR) < 0.05. (**D**) Quantification of GSDMD protein levels by immunoblotting in different tissues of WT (C57BL/6N) mice (*n =* 3). (**E** and **F**) Representative immunoblotting (**E**) and quantification (**F**) of left ventricular tissues from mice subjected to AMI for different time points (1 day, 3 days, 5 days) or sham surgery (*n =* 5–6 per group). β-Tubulin or HSP90 was used as a loading control. Data are presented as mean ± SD and were analyzed by 1-way ANOVA with Tukey’s correction for multiple comparisons (**F**). *P < 0.05; **P < 0.01; ***P < 0.001; ****P < 0.0001. NS, not significant.

We initially examined transcription levels of key factors involved in the acute inflammatory response to AMI by RNA sequencing of murine heart samples after AMI. Hierarchical clustering demonstrated samples from different experimental groups (sham, 1-day, and 7-day post-AMI groups) to be well separated from each other, while biological replicates from the same group clustered together well (Supplemental Figure 1A; supplemental material available online with this article; https://doi.org/10.1172/JCI151268DS1). We found that 204 genes were significantly upregulated in day 1 AMI samples compared with the sham samples and day 7 AMI samples, while 64 genes were significantly downregulated (Figure 1A). Differentially expressed genes (DEGs) that had consistent expression patterns across sham, day 1 AMI, and day 7 AMI were further clustered together, and genes encoding the components of the inflammasome showed a distinct increase, indicating the inflammasome may be activated after AMI (Figure 1, B and C, and Supplemental Figure 1). Furthermore, Gene Ontology (GO) “Biological Process” enrichment analysis of DEGs also suggested upregulation of inflammatory responses, including “neutrophil chemotaxis” and “cellular response to IL-1” (Supplemental Figure 1C). Interestingly, *Nlrp3*, which encodes a pattern recognition receptor, was rapidly upregulated within 24 hours after AMI, followed by a decrease in expression on day 7 after AMI (Figure 1C). The expression of the downstream components of NLRP3 activation, including *Casp1*, *Gsdmd*, and *Il1b*, was upregulated in response to AMI, indicating that the myocardial NLRP3 inflammasome could be activated during AMI (Figure 1C). We further validated these findings by evaluating the protein level and activation level of *Gsdmd* in the heart. GSDMD exhibited a low level of expression in heart but high levels in other tissues like the intestine, liver, and spleen (Figure 1D), consistent with previous reports that GSDMD exhibited high levels in immunocytes and fibroblasts and low levels in cardiomyocytes, endothelial cells, and smooth muscle cells (19, 20). Intriguingly, both expression and activation of GSDMD in heart were remarkably enhanced in response to myocardial ischemia/infarction in left anterior descending (LAD) ligation–operated wild type (WT) mice, as early as 24 hours after AMI (Figure 1, E and F). In addition, NLRP3, caspase-1 cleavage, and particularly IL-1β were induced early during myocardial ischemia/infarction (Figure 1, E and F). Taken together, these data suggest that the NLRP3 inflammasome and GSDMD-induced pyroptosis may be activated early after AMI and the likely source is from infiltrating leukocytes.

### Loss of GSDMD attenuates myocardial injury after AMI.

**Figure 2:**
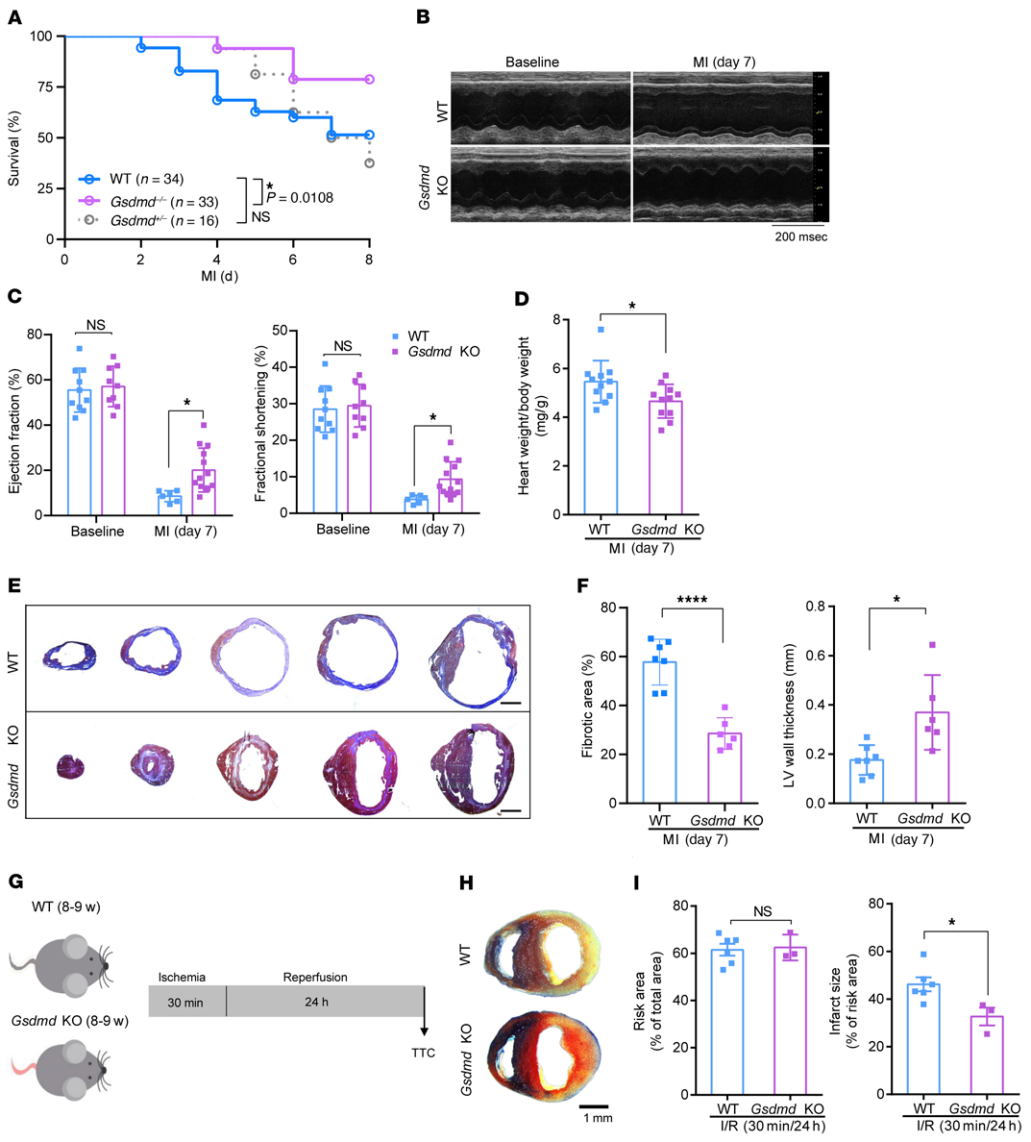
Loss of GSDMD attenuates myocardial injury after AMI. (**A**) Kaplan-Meier survival curves comparing post-AMI survival of WT (C57BL/6N) mice (*n =* 34) to that of *Gsdmd*^−/−^ mice (*n =* 33) or *Gsdmd*^+/−^ mice (*n =* 16). Statistical significance was determined by Mantel-Cox test. (**B** and **C**) Echocardiography images (**B**) and M-mode quantification (**C**) of ejection fraction (left) and fractional shortening (right) for WT or *Gsdmd*^−/−^ mice before or 1 week after AMI (baseline: WT, *n =* 10; *Gsdmd*^−/−^, *n =* 9; 1 week: WT, *n =* 6; *Gsdmd*^−/−^, *n =* 12). (**D**) A comparison of heart weight/body weight ratio between WT mice and *Gsdmd*^−/−^ mice 1 week after AMI (WT, *n =* 11; *Gsdmd*^−/−^, *n =* 11). (**E** and **F**) Masson’s trichrome staining (**E**) and quantification of fibrotic area and left ventricular (LV) wall thickness (**F**) of short-axis heart sections from WT or *Gsdmd*^−/−^ mice 1 week after AMI (WT, *n =* 7; *Gsdmd*^−/−^, *n =* 6). Scale bar: 1 mm. (**G**) Schematic diagram showing the ischemia/reperfusion (I/R) surgery strategy for WT and *Gsdmd*^−/−^ mice. (**H** and **I**) Representative images of Evans blue dye and triphenyltetrazolium chloride (TTC) staining (**H**) and quantification of risk area (left) and infarct size (right) (**I**) for WT or *Gsdmd*^−/−^ mice after I/R surgery (WT, *n =* 6; KO, *n =* 3). Data are presented as mean ± SD. *P < 0.05; ****P < 0.0001, as analyzed by 1-way ANOVA followed by Bonferroni’s multiple comparison test (**C**) or unpaired, 2-tailed Student’s *t* test (**D**, **F**, and **I**). NS, not significant.

To determine the overall role of GSDMD in AMI, we initially utilized *Gsdmd*^−/−^ (global knockout) mice and subjected the hearts to sham operations or AMI (as will be described later, bone marrow transplantation and leukocyte depletion studies will provide tissue-specific knockout studies). Unexpectedly, we observed significantly improved survival of the *Gsdmd*^−/−^ mice after AMI compared with AMI-operated WT littermate controls (78.7% versus 50%, *P* = 0.0108; Figure 2A). *Gsdmd* deficiency appeared to significantly improve left ventricular (LV) systolic function, which was severely impaired following AMI in WT littermate controls (Figure 2, B and C). Consistently, the heart weight to body weight ratio in *Gsdmd*^−/−^ mice was significantly decreased compared with that of the littermate controls (Figure 2D). We further determined the effect of *Gsdmd* deficiency on cardiac structural remodeling after AMI. Masson’s trichrome staining analysis and quantification of the scar showed that the hearts from *Gsdmd*^−/−^ mice had significant reduction in fibrotic scar size (Figure 2, E and F) and increase in thickness of the LV wall (Figure 2F). Apart from utilizing LAD ligation–operated mice, we also compared the myocardial infarction size in response to I/R (30 minutes/24 hours) in mice (Figure 2G). Remarkably, we observed that *Gsdmd*^−/−^ mice also exhibited a significant reduction in infarct size compared with that of the littermate controls (Figure 2, H and I). These data suggested that *Gsdmd* deficiency reduces infarct size, preserves cardiac function, and improves survival after AMI. We set forth to determine the mechanisms for this unexpected protective effect.

### GSDMD is essential for recruitment of neutrophils/monocytes to the infarcted heart.

**Figure 3:**
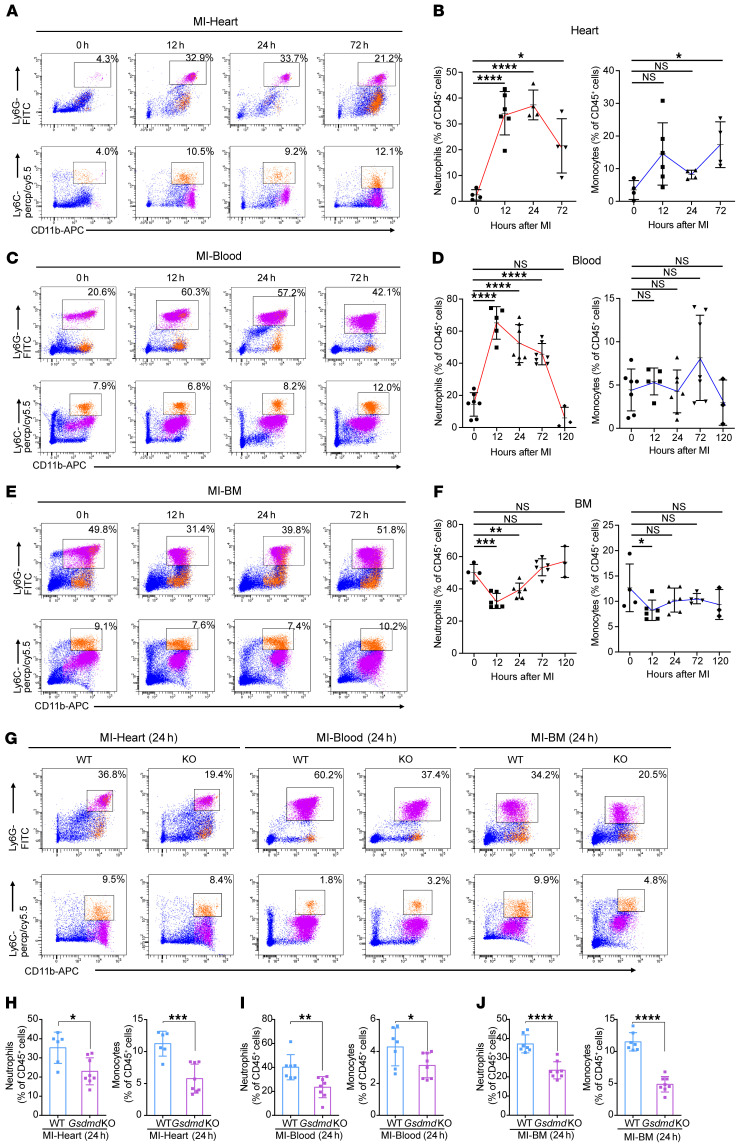
GSDMD is essential for recruitment of neutrophils/monocytes to the AMI heart. (**A**–**F**) Flow cytometric analysis and quantification of Cd11b^+^Ly6G^+^ neutrophils and Cd11b^+^Ly6C^+^ monocytes in heart (**A**), blood (**C**), or bone marrow (BM) (**E**) from WT or *Gsdmd*^−/−^ mice at different time points (12 hours, 24 hours, 72 hours) after AMI or sham surgery (*n =* 4–7), along with their quantification (**B**, **D**, and **F**). (**G**–**J**) Flow cytometric analysis and quantification of Cd11b^+^Ly6G^+^ neutrophils and Cd11b^+^Ly6C^+^ monocytes in heart (**H**), blood (**I**), or BM (**J**) from WT or *Gsdmd*^−/−^ mice 24 hours after AMI (*n =* 7–8). Data are presented as mean ± SD. *P < 0.05; **P < 0.01; ***P < 0.001; ****P < 0.0001, as analyzed by 1-way ANOVA followed by Bonferroni’s multiple comparison test (**B**, **D**, and **F**) or unpaired, 2-tailed Student’s *t* test (**H**–**J**). NS, not significant.

**Figure 4:**
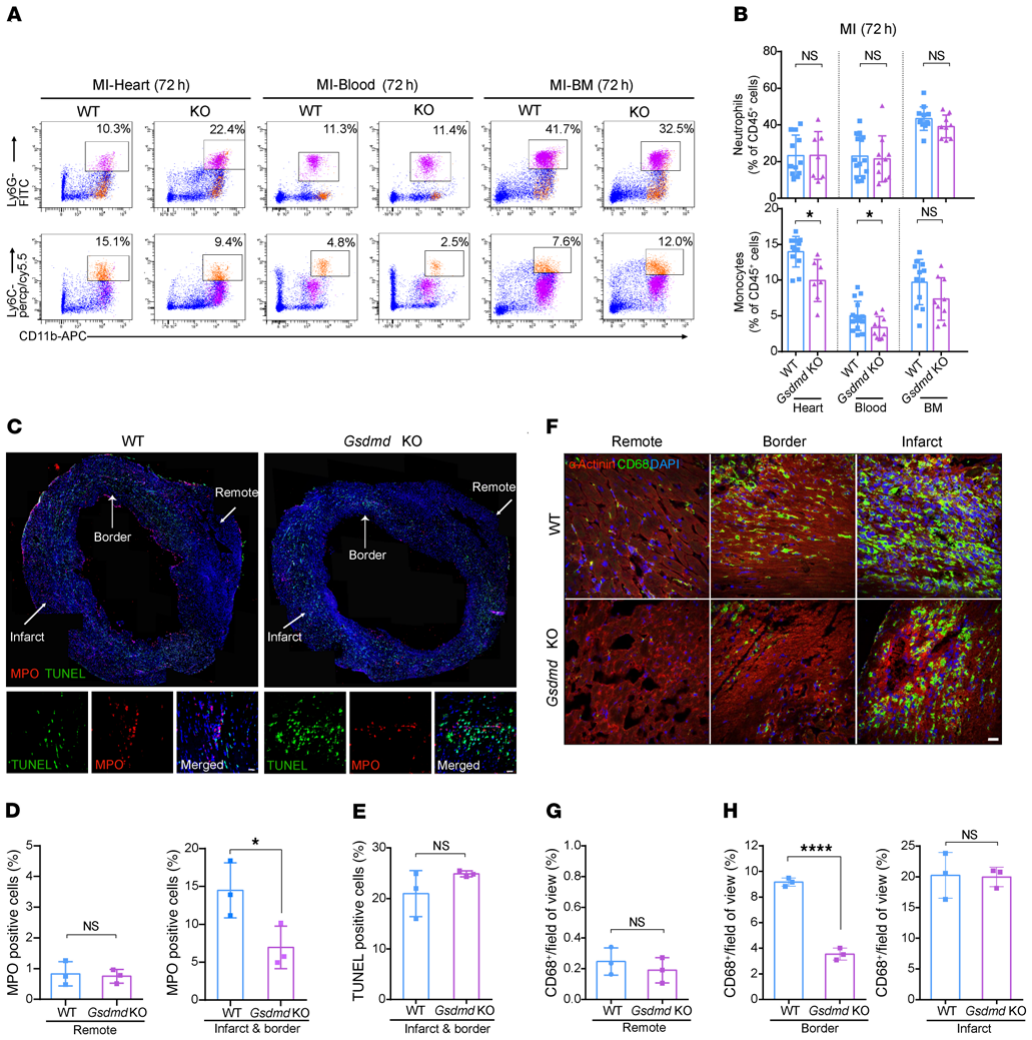
GSDMD is essential for recruitment of neutrophils/monocytes to the infarcted heart. (**A** and **B**) Flow cytometric analysis and quantification of Cd11b^+^Ly6G^+^ neutrophils and Cd11b^+^Ly6C^+^ monocytes in the heart (left), blood (middle), or BM (right) from WT or *Gsdmd*^−/−^ mice 72 hours after AMI (*n =* 7–15). (**C**) Immunofluorescence imaging and magnification for MPO (red), TUNEL (green), and DAPI (blue) on heart sections from WT or *Gsdmd*^−/−^ mice 24 hours after AMI. Scale bar: 20 μm. (**D** and **E**) Quantification of ratios of MPO^+^ or TUNEL^+^ cells of heart sections from WT or *Gsdmd*^−/−^ mice. Each value was averaged from the values of 7 fields of view from the same mouse (*n =* 3 per group). (**F**) Immunofluorescence imaging of heart sections from WT or *Gsdmd*^−/−^ mice 3 days after AMI showing α-actinin (red), CD68 (green), and DAPI (blue). Representative fields of remote zone, border zone, and infarct zone are presented. Scale bar: 20 μm. (**G** and **H**) Quantification of CD68^+^ area proportion in the field of view in remote zone (**G**) and border and infarct zones (**H**) of heart sections from WT or *Gsdmd*^−/−^ mice. Each value was averaged from the values of 5 fields of view from the same mouse (*n =* 3 per group). Data are presented as mean ± SD. *P < 0.05; ****P < 0.0001 by multiple 2-tailed Student’s *t* test (**B**) or unpaired, 2-tailed Student’s *t* test (**D**, **E**, **G**, and **H**). NS, not significant.

To explore the mechanisms underlying GSDMD deficiency–conferred cardioprotection after AMI, and based on recent suggestions that GSDMD may play a role in neutrophil production/mobilization, we investigated leukocyte infiltration and leukocytosis in AMI (Supplemental Figure 2). After AMI, an increase particularly in neutrophil (and to a lesser extent monocyte) recruitment to the infarcted heart occurred within 12 hours, peaking at 24 hours (neutrophils) and 72 hours (monocytes) during the 3-day observation period (Figure 3, A and B). The number of neutrophils (and monocytes) in the blood exhibited a similar pattern, with neutrophils peaking at 12 hours (Figure 3, C and D). This initial surge in blood neutrophils might result from mass exodus of neutrophils from the hematopoietic stem and progenitor cells in the bone marrow (Figure 3, E and F). Consistent with this notion, there was an initial significant decrease in the overall number of neutrophils (and monocytes) in the bone marrow followed by an increase in numbers (Figure 3, E and F), supporting the majority of the initial neutrophil mobilization as being from the bone marrow followed by new production of neutrophils, consistent with previous reports (16). Furthermore, in *Gsdmd*^−/−^ mice, the observed increase compared with WT was significantly reduced in the heart, blood, and bone marrow 24 hours after AMI (Figure 3, G–J), supporting an important role for GSDMD in neutrophil generation and mobilization in AMI. Intriguingly, there was no difference in the number of neutrophils in the heart, blood, and bone marrow between WT and *Gsdmd*^−/−^ mice 72 hours after AMI (Figure 4, A and B). In contrast, *Gsdmd*^−/−^ mice exhibited a marked reduction in the number of monocytes both in the heart and blood 72 hours after AMI compared with WT mice (Figure 4, A and B). These results suggest that the inhibition of GSDMD could apply a brake on neutrophil mobilization at the initial stages of the inflammatory response to an AMI.

To validate these findings, we further sectioned the infarcted heart followed by immunofluorescence staining with anti-myeloperoxidase (anti-MPO) or -CD68 antibodies to specifically label and visualize the local neutrophils (MPO^+^) and monocytes/macrophages (CD68^+^). At 24 hours after AMI, in the WT mice a large number of MPO^+^ neutrophils were recruited to the infarcted heart, while the number of neutrophils in the infarcted heart of *Gsdmd*^−/−^ mice was markedly reduced (Figure 4, C and D). Notably, there was no difference in the number of the TUNEL-positive apoptotic cells in the infarcted heart between *Gsdmd*^−/−^ mice and controls (Figure 4, C and E), suggesting that GSDMD deletion did not affect cardiac apoptosis after AMI, consistent with its key specific role in pyroptosis. By 72 hours after AMI, a large number of monocytes/macrophages were also recruited into both the infarct zone and border zone of the heart in WT mice (Figure 4, F–H). Again, the *Gsdmd*^−/−^ mice showed a significant decrease in monocyte/macrophage infiltration, particularly in the infarct border zone (Figure 4, G and H), suggesting the critical role of GSDMD in mediating function of monocytes/macrophages in the later acute phase of an AMI. Together, the data support the idea that GSDMD is involved in the recruitment of neutrophils (and monocytes) to the infarcted heart, contributing to the inflammatory response.

**Figure 5:**
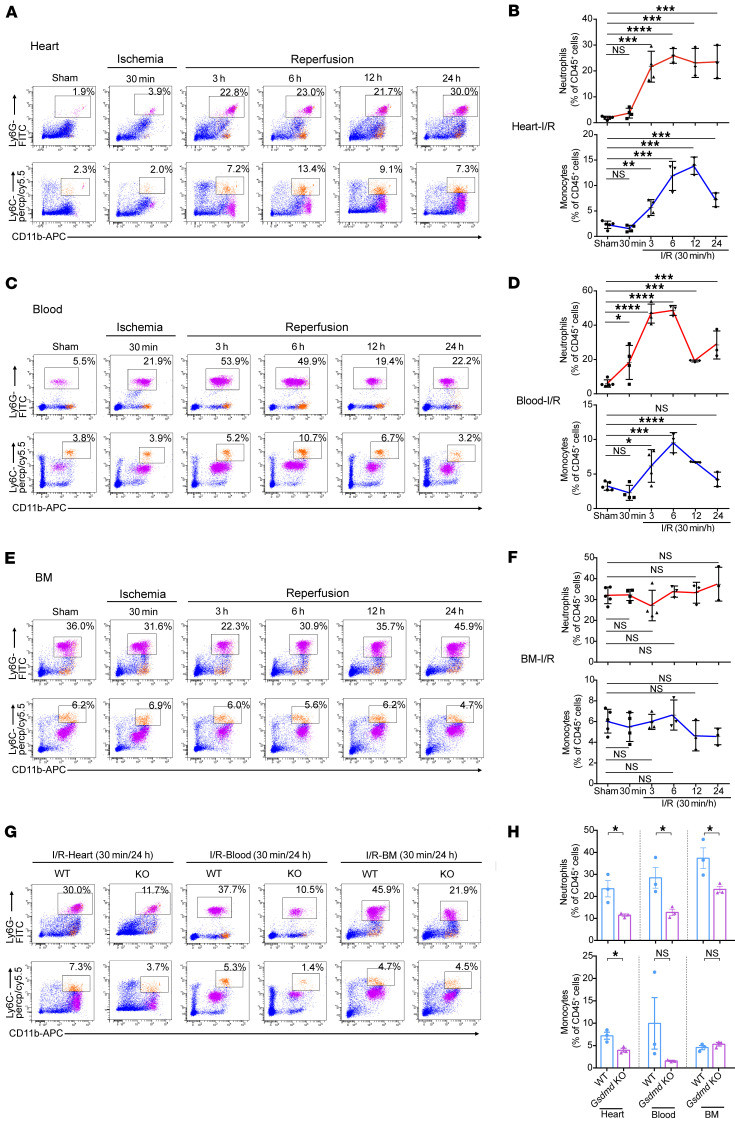
GSDMD is essential for recruitment of neutrophils/monocytes to the I/R heart. (**A**–**F**) Flow cytometric analysis and quantification of Cd11b^+^Ly6G^+^ neutrophils and Cd11b^+^Ly6C^+^ monocytes in heart (**A** and **B**), blood (**C** and **D**), or bone marrow (BM) (**E** and **F**) from WT or *Gsdmd*^−/−^ mice at different reperfusion time points (3 hours, 6 hours, 12 hours, 24 hours) after I/R or sham surgery. Corresponding *n* values are indicated in the plots. The statistical significance of sham versus 3, 6, 12, or 24 hours is indicated (*n =* 3–5). (**G** and **H**) Flow cytometric analysis (**G**) and quantification (**H**) of Cd11b^+^Ly6G^+^ neutrophils and Cd11b^+^Ly6C^+^ monocytes in heart (left), blood (middle), or BM (right) from WT or *Gsdmd*^−/−^ mice 24 hours after I/R (*n* = 3). Data are presented as mean ± SD. *P < 0.05; **P < 0.01; ***P < 0.001; ****P < 0.0001 by 1-way ANOVA followed by Bonferroni’s multiple-comparison test (**B**, **D**, and **F**) or multiple 2-tailed Student’s *t* test (**H**). NS, not significant.

In addition, similar patterns of neutrophil and monocyte counts were observed in response to myocardial I/R (Figure 5, A–F). Notably, *Gsdmd* deletion did not affect baseline leukocyte proportion (Supplemental Figure 3). Collectively, observations of a significant reduction in the mobilized neutrophils in the heart 24 hours after AMI (Figure 3, G–J) and I/R (Figure 5, G and H) in *Gsdmd*^−/−^ mice suggest a distinct role of GSDMD in regulating neutrophils in response to AMI.

### GSDMD deficiency reduces cell death and IL-1β.

**Figure 6:**
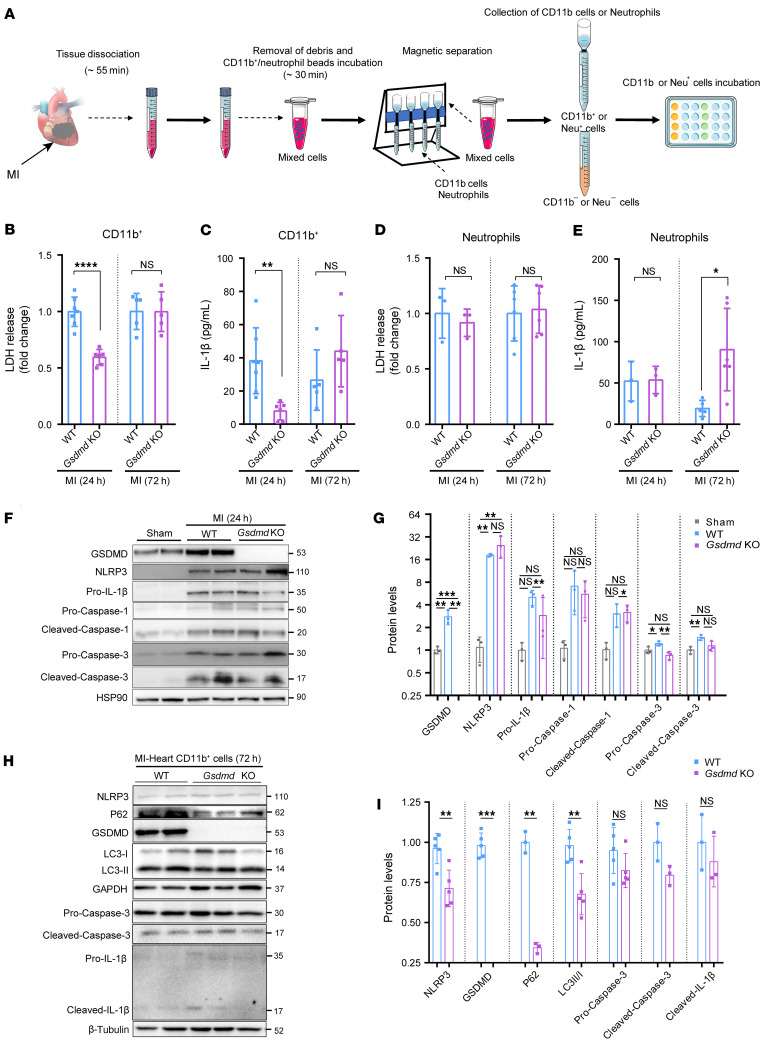
GSDMD deficiency suppresses cell death and IL-1β secretion. (**A**) Schematic diagram showing the strategy of preparing samples for IL-1β and lactate dehydrogenase (LDH) detection in Cd11b^+^ myeloid-derived cells and neutrophils from the heart. Neu^+^, neutrophils; Neu^–^, neutrophil free. (**B**) Secretion levels of LDH from leukocytes from the heart of WT or *Gsdmd*^−/−^ mice 24 hours and 72 hours after AMI. (**C**) Production of IL-1β from Cd11b^+^ cells from the heart of WT or *Gsdmd*^−/−^ mice 24 hours and 72 hours after AMI assessed by ELISA. The corresponding *n* values are indicated in the plot. (**D**) Secretion levels of LDH from neutrophils isolated from the heart of WT or *Gsdmd*^−/−^ mice 24 hours and 72 hours after AMI. (**E**) Production of IL-1β from neutrophils isolated from the heart of WT or *Gsdmd*^−/−^ mice 24 hours and 72 hours after AMI assessed by ELISA. The corresponding *n* values are indicated in the plot. (**F** and **G**) Representative immunoblotting images (**F**) and quantification (**G**) of protein levels in heart left ventricular tissues from WT or *Gsdmd*^−/−^ mice 24 hours after AMI or sham surgery (*n =* 3 per group). (**H** and **I**) Representative immunoblotting images (**H**) and quantification (**I**) of protein levels of heart Cd11b^+^ cells from WT or *Gsdmd*^−/−^ mice 72 hours after AMI or sham surgery (*n =* 3–5). Data are presented as mean ± SD. *P < 0.05; **P < 0.01; ***P < 0.001; ****P < 0.0001, as analyzed by unpaired, 2-tailed Student’s *t* test (**B**–**E** and **I**) or 1-way ANOVA with Tukey’s correction for multiple comparisons (**G**). NS, not significant.

As noted above, there was no difference in TUNEL-positive apoptotic cell death between the WT and *Gsdmd*^–/–^ mice. AMI-induced activation of the NLRP3 inflammasome triggers further myocardial damage indirectly through the release of IL-1β and directly through promotion of inflammatory cell death via pyroptosis (8). Given GSDMD’s role in releasing IL-1β from neutrophils independently of pore formation (18), we next sought to explore possible mechanisms for reduced recruitment of neutrophils to the infarcted heart caused by GSDMD deficiency. To determine secretion of IL-1β from leukocytes in the infarcted heart, CD11b^+^ leukocytes or Ly6G^+^ neutrophils were isolated from sham and ischemic mouse hearts (*Gsdmd*^−/−^ mice and littermate controls), as described previously (21). The isolated CD11b^+^ leukocytes or Ly6G^+^ neutrophils were cultured for 24 hours, followed by assays to measure lactate dehydrogenase (LDH), IL-1β, IL-18, and monocyte chemoattractant protein 1 (MCP-1) levels (Figure 6A and Supplemental Figure 4, A and B). We observed significant reduction in both LDH and IL-1β levels in isolated CD11b^+^ leukocytes from *Gsdmd*^−/−^ mice compared with those from littermate controls 24 hours after AMI, with no significant difference 72 hours after AMI (Figure 6, B and C). In contrast, there was no significant difference in both IL-1β and LDH levels in isolated neutrophils from *Gsdmd*^−/−^ mice and WT mice 24 hours after AMI, but a significant increase in IL-1β level in *Gsdmd*^−/−^ mice 72 hours after AMI (Figure 6, D and E, and Supplemental Figure 4, C and D). Consistently, serum LDH level was markedly elevated 24 hours after AMI in WT mice, while it was significantly reduced in *Gsdmd*^−/−^ mice (Supplemental Figure 4E). However, serum IL-1β was detected at equal levels in WT mice and *Gsdmd*^−/−^ mice 72 hours after AMI (Supplemental Figure 4F). These results suggest that it is neutrophil-released IL-1β that dominates the serum IL-1β level 72 hours after AMI.

To explore how GSDMD modulates neutrophil death and IL-1β release independently of plasma membrane GSDMD pores and pyroptosis, we further analyzed the isolated heart and leukocyte samples from *Gsdmd*^−/−^ mice compared to those from littermate controls 24 and 72 hours after AMI. Although there was no significant difference in NLRP3 activation and cleavage of caspase-1 and pro-IL-1β in hearts 24 hours after AMI between WT and *Gsdmd*^−/−^ mice (Figure 6, F and G), cleaved LC3 (autophagy marker) in CD11b^+^ leukocytes or Ly6G^+^ neutrophils samples from the *Gsdmd*^−/−^ mice 72 hours after AMI was significantly increased compared with that in the WT mice (Figure 6, H and I, and Supplemental Figure 4, G–I). These results indicated that the autophagic flux in neutrophils may be enhanced by GSDMD deficiency, contributing to the release of IL-1β from neutrophils. Taken together, these data suggest that AMI-mediated activation of GSDMD results in the release of IL-1β, possibly leading to cardiac inflammation by recruitment of neutrophils to the infarcted heart.

### GSDMD-dependent bone marrow–derived myeloid cells contribute to acute inflammatory response.

**Figure 7:**
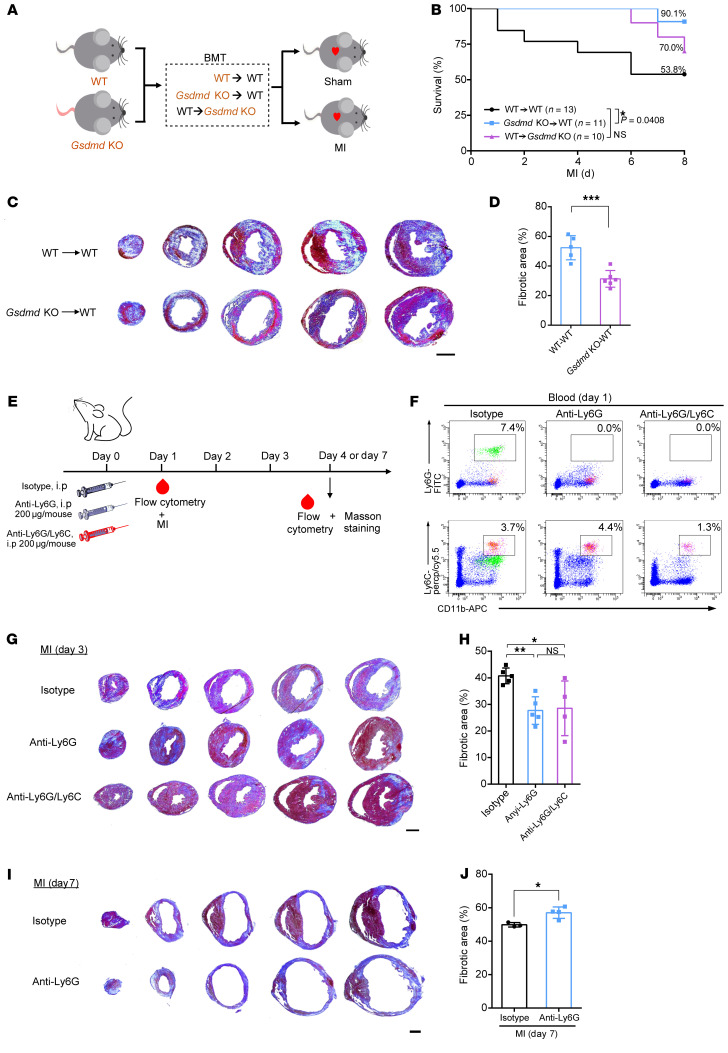
GSDMD-dependent bone marrow–derived myeloid cells contribute to acute inflammatory responses. (**A**) Schematic diagram showing the strategy of the bone marrow transplantation (BMT) experiment. (**B**) Kaplan-Meier survival curves comparing post-AMI survival of WT → WT mice (*n =* 13) to that of *Gsdmd-*KO → WT mice (*n =* 11) or WT → *Gsdmd*-KO mice (*n =* 10). Statistical significance was determined by Mantel-Cox test. (**C** and **D**) Masson’s trichrome staining (**C**) and quantification (**D**) of fibrotic area of short-axis heart sections from WT → WT (*n =* 5) or *Gsdmd-*KO → WT (*n =* 6) mice 3 days after AMI. Scale bar: 1 mm. (**E**) Schematic diagram showing the strategy for neutrophil and monocyte depletion. (**F**) Flow cytometric gating of Ly6G^+^ neutrophils and Ly6C^+^ monocytes validating the successful elimination of neutrophils or monocytes in mice. (**G** and **H**) Masson’s trichrome staining (**G**) and quantification (**H**) of fibrotic area of short-axis heart sections from mice treated with isotype IgG (*n =* 5), anti-Ly6G antibody (*n =* 5), or anti-Ly6G/Ly6C antibody (*n =* 4) 3 days after AMI. Scale bar: 1 mm. (**I** and **J**) Masson’s trichrome staining (**I**) and quantification (**J**) of fibrotic area of short-axis heart sections from mice treated with isotype IgG (*n =* 3) or anti-Ly6G antibody (*n =* 4) 1 week after AMI. Scale bar: 1 mm. Data are presented as mean ± SD. *P < 0.05; **P < 0.01; ***P < 0.001 by unpaired, 2-tailed Student’s *t* test (**D** and **J**) or 1-way ANOVA followed by Tukey’s multiple-comparison test (**H**). NS, not significant.

Given that release of IL-1β induced by myocardial injury can be from leukocytes and non-leukocytes (e.g., fibroblasts) (10), we hypothesized that AMI-induced neutrophil infiltration requires GSDMD activation in cardiac neutrophils. To test this hypothesis, we transplanted bone marrow from WT or *Gsdmd*^−/−^ mice into WT mice, or bone marrow from WT into *Gsdmd*^−/−^ mice, and then subjected the transplanted animals to AMI (Figure 7A and Supplemental Figure 5A). This serves as a bone marrow–specific knockout of *Gsdmd*. Consistent with our proposed sequelae of events, AMI-induced poor survival and adverse cardiac remodeling were improved by *Gsdmd−/−* bone marrow transplantation (Figure 7, B–D). There was no difference in cardiac function 1 week after MI among those that survived (Supplemental Figure 5, B–F). Given that transplantation of WT bone marrow into *Gsdmd*^−/−^ mice did not fully restore the WT phenotype (Figure 7B), a number of explanations are possible, including that GSDMD in some nonradiosensitive cells may also contribute.

To further characterize the role of neutrophils in contributing to myocardial injury in vivo, we next depleted neutrophils or neutrophils/monocytes by intraperitoneal injection of anti-Ly6G and anti-Ly6G/Ly6C antibodies, respectively (Figure 7E). Injection of anti-Ly6G antibody effectively depleted the circulating neutrophils and did not affect the number of monocytes (Figure 7F and Supplemental Figure 6). Furthermore, anti-Ly6G/Ly6C injection led to an effective clearance of circulating neutrophils and monocytes (Figure 7F). Both groups of mice, i.e., those with neutrophil depletion and those with neutrophil/monocyte clearance, exhibited a significantly reduced infarct size compared with control mice (Figure 7, G and H). Importantly, there was no significant difference in infarct size between anti-Ly6G–injected mice and anti-Ly6G/Ly6C–injected mice 72 hours after AMI (Figure 7H), which emphasized the critical role of neutrophils in promoting myocardial injury. However, depletion of neutrophils with anti-Ly6G for 1 week mildly increased infarct size after AMI (Figure 7, I and J). This key result has important therapeutic implications, suggesting neutrophil depletion should be short term (first 3 days), as longer-term depletion (1 week) can be detrimental. These data suggest that bone marrow–derived neutrophils contribute to acute inflammatory responses to AMI and their conferred cardioprotection depends on GSDMD activity.

### Pharmacological inhibition of GSDMD reduces infarct size after AMI.

**Figure 8:**
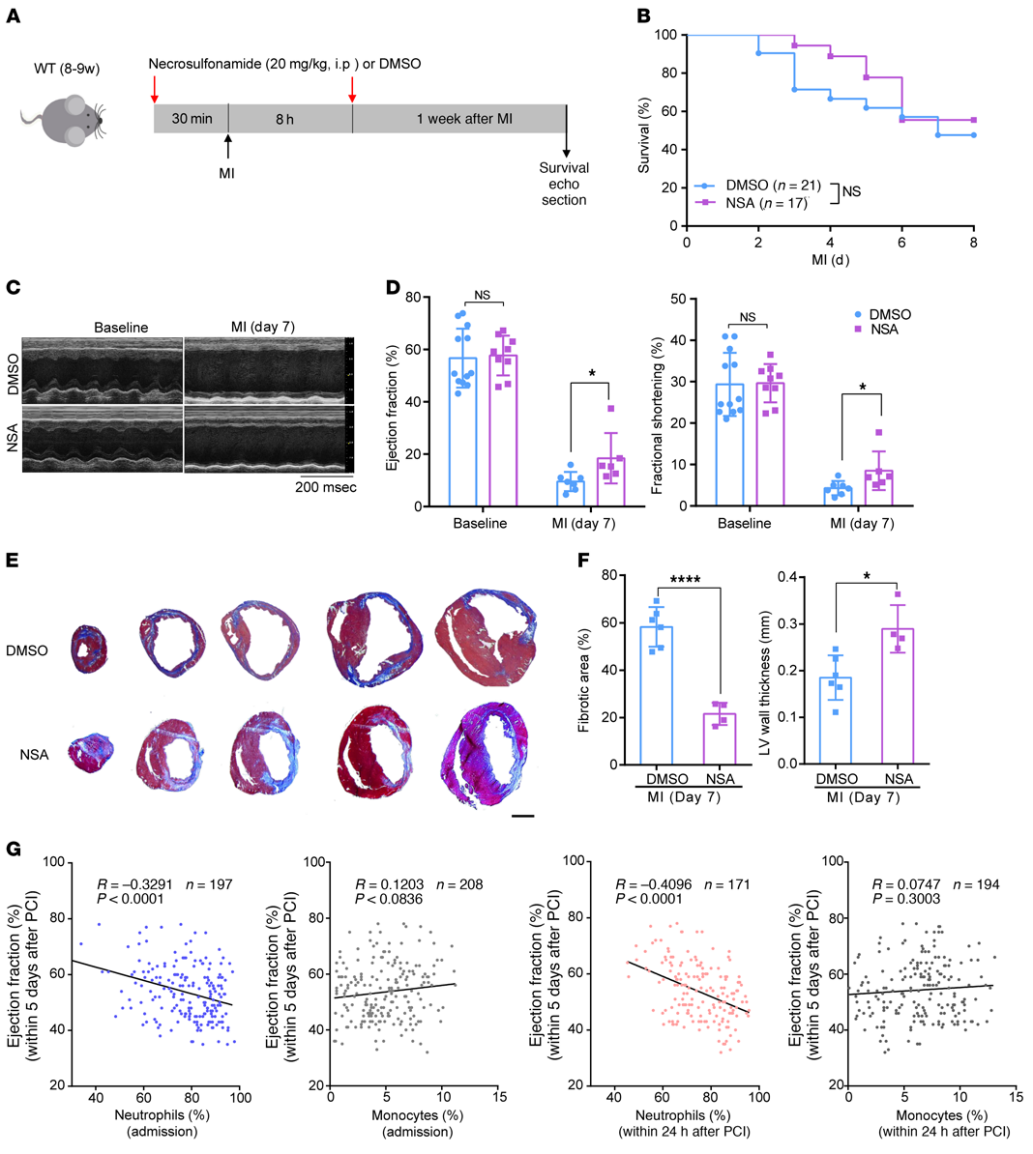
Pharmacological inhibition of GSDMD reduces infarct size after AMI. (**A**) Schematic diagram showing the strategy of NSA administration to the mice. (**B**) Kaplan-Meier survival curves comparing post-AMI survival of control (DMSO administration) mice (*n =* 21) to that of mice administered NSA (*n =* 17). Significance was determined by Mantel-Cox test. (**C** and **D**) Echocardiography images (**C**) and M-mode quantification (**D**) of ejection fraction (left) and fractional shortening (right) for control mice or mice with NSA administration before or 1 week after AMI (baseline: DMSO, *n =* 12; NSA, *n* = 9; 1 week: DMSO, *n =* 7; NSA, *n =* 6). (**E** and **F**) Masson’s trichrome staining (**E**) and quantification of fibrotic area and left ventricular (LV) wall thickness (**F**) of short-axis heart sections from control mice or mice with NSA administration 1 week after AMI (DMSO, *n =* 6; NSA, *n =* 4). Scale bar: 1 mm. (**G**) Analysis of correlation between ejection fraction of AMI patients within 5 days after PCI and the percentage of neutrophils or monocytes in peripheral blood at the point of admission (left 2 graphs) or in the patients within 24 hours after PCI was performed (right 2 graphs) with Pearson’s correlation test. Data are presented as mean ± SD. *P < 0.05; ****P < 0.0001 by 1-way ANOVA with Tukey’s correction for multiple comparisons (**D**) or unpaired, 2-tailed Student’s *t* test (**F**). NS, not significant.

The bone marrow transplant data suggest that GSDMD inhibition reduces infarct size and preserves cardiac function through leukocyte suppression. Given that pyroptotic cell death can be pharmacologically inhibited by necrosulfonamide (NSA) (22), the role of NSA as a therapy in the initial inflammatory response was then tested in vivo in the above mouse model of permanent ligation of the LAD (Figure 8A and Supplemental Figure 7A). We optimized the dosage of NSA for in vivo stability, according to previous reports (22), and found that NSA administration at a dose of 20 mg/kg either 30 minutes before LAD ligation (Figure 8) or within 30 minutes (DMSO: 11.3 ± 1.9 minutes; NSA 11.2 ± 2.2 minutes) after LAD ligation (Supplemental Figure 7) did not demonstrate any adverse short-term survival effect (toxicity or arrhythmia) 1 week after AMI (Figure 8B and Supplemental Figure 7B). However, there was significant improvement in systolic function (Figure 8, C and D, and Supplemental Figure 7, C and D). Masson’s trichrome staining analysis of the scar showed that NSA treatment significantly reduced the fibrotic scar size (Figure 8E and Supplemental Figure 7, E and F) and increased LV wall thickness (Figure 8F). Based on our findings from the murine AMI models, GSDMD inhibition (within hours of AMI) may be a novel therapy to reduce scar formation and prevent heart failure after AMI. Inhibition of excess early (1–3 days) leukocyte mobilization and myocardial leukocyte infiltration may also be a potential strategy for therapy of AMI.

### Human studies confirm the importance of AMI-associated neutrophilia.

To highlight the impact of post-AMI neutrophilia in human subjects, we recruited 234 patients who had an AMI with only a single LAD branch blockage (analogous to our mouse LAD ligation model; Supplemental Table 1) and correlated their neutrophil percentage to ejection fraction 5 days after percutaneous coronary intervention (PCI). There was a clear statistical negative correlation (*R* = –0.41, *P* < 0.0001; Figure 8G), with increased neutrophil percentage (greater than 60% indicates neutrophilia) being associated with a reduced ejection fraction. In contrast, there was no significant correlation between monocytes (both admission and within 24 hours after PCI) and ejection fraction within 5 days after PCI. Taken together, our mouse AMI studies in combination with our preliminary human studies highlight the potential benefits of GSDMD inhibition in improving ventricular function and survival after AMI.

## Discussion

Despite significant advances in percutaneous and surgical reperfusion, many patients who have an AMI ultimately develop heart failure with its associated poor prognosis. New mechanism-based therapies are urgently needed. An intense inflammatory response is triggered after myocardial ischemia and necrosis (23). Inflammation, although essential for wound healing, can mediate excessive scar formation and dysfunctional ventricular remodeling (24, 25). Clinically, neutrophilia (a key component of the inflammatory response to AMI) correlates with major adverse cardiovascular events in patients with AMI (16), implying neutrophil reduction may have more favorable outcomes. However, the mechanisms that determine neutrophil generation and recruitment to the infarcted heart remain unclear. Herein, we report that AMI-induced neutrophilia and early neutrophil infiltration into the heart are linked to increased expression and activation of inflammasome-effector GSDMD. Global knockout of *Gsdmd*, bone marrow–specific knockout of *Gsdmd* by bone marrow transplantation, and chemical inhibition of GSDMD reduced infarct size and improved cardiac function after AMI. In addition, GSDMD deficiency attenuated the myocardial injury in a murine I/R model. Loss of GSDMD resulted in decreased early generation and mobilization of neutrophils and monocytes to the infarcted heart. Furthermore, clearance of neutrophils in vivo improved the heart function after AMI. Taken together, our findings support the notion that inhibition of early neutrophil generation and mobilization is cardioprotective for AMI.

Cardiac neutrophils are the first responder in amplifying the acute inflammatory response after AMI. The initial wave of infiltrating neutrophils sets the tone for the ensuing inflammatory response by releasing key factors that activate the NLRP3 inflammasome, and promote the secretion of IL-1β (7). The released IL-1β interacts with IL-1 receptor type 1 on myeloid progenitor cells in the bone marrow and stimulates granulopoiesis in a cell-autonomous manner. Genetic deletion or pharmacological inhibition of the NLRP3 inflammasome/IL-1β signaling axis dampens granulopoiesis and improves cardiac function in mouse models of AMI (8). Although GSDMD promotes IL-1β release from hyperactive macrophages (26) and targeting IL-1β reduces leukocyte production after AMI (7, 11), how the release of IL-1β from distinct subsets of leukocytes and non-leukocytes is regulated in response to AMI is not fully understood. Our study demonstrated that GSDMD was predominantly expressed in leukocytes, but not in other types of cells in the heart tissue (data not shown). Importantly, through bone marrow transplantation, we demonstrated that GSDMD-dependent neutrophil recruitment was required for myocardial injury in the early phase. GSDMD deficiency reduced release of IL-1β from neutrophils and the acute inflammatory response after AMI. For clinical translation, we further tested an inhibitor of pyroptotic cell death, NSA, which has recently been reported to inhibit GSDMD-mediated pore formation in cell membranes and subsequent pyroptosis (22) in a murine AMI model. We demonstrated that pharmacological inhibition of GSDMD also conferred cardioprotection after AMI by reducing scar size and enhancing heart function. Although there is no difference in survival, significantly increased ejection fraction would be clinically associated with improved signs and symptoms of heart failure, improved exercise tolerance, as well as reduced hospitalizations. With an increased cohort of treated mice, as well as longer-term follow-up, we anticipate that the significantly improved ejection fraction will translate to increased survival. These findings highlight the potential therapeutic application for targeting GSDMD early after AMI.

There is a contradictory study showing that neutrophil depletion has no effect on infarct size 24 hours after AMI, and also progressively worsens cardiac function from day 7 to day 14 (27). Since neutrophils are necessary in the repair process, optimizing the dosage and timing of anti-Ly6G injection is essential in addition to other supportive genetic, neutrophil depletion, and bone marrow transplant data. This suggests that the timing of interventional strategies for targeting GSDMD as well as the degree of neutrophil inhibition are critical in preventing postinfarction heart failure. Previous in vitro studies demonstrated that in the absence of the pyroptosis-mediating substrate GSDMD, caspase-1 activates caspase-3 and induces apoptosis (28), indicating a possible bidirectional crosstalk between apoptosis and pyroptosis in monocytes and macrophages (29). However, in the present study, in vivo analysis with Western blotting and TUNEL staining revealed that loss of GSDMD did not significantly change the overall level of apoptosis in the infarcted heart (Figures 4C and 6F). One recent in vitro study using ATG7-deficient cells demonstrated that neutrophils secrete IL-1β through N-terminal GSDMD trafficking to neutrophil organelles, an autophagy-dependent mechanism (18), consistent with our findings that GSDMD regulates IL-1β release independently of plasma membrane pores and pyroptosis in neutrophils. In contrast, we have now demonstrated that GSDMD deficiency triggered autophagic flux in neutrophils using ex vivo assays (Figure 6, H and I, and Supplemental Figure 4). This will require further detailed exploration.

There are several limitations to this study, including choice of the infarct model. To establish the infarct model, we applied permanent ligation of a normal coronary artery, which differs substantially from the process of atherothrombosis in humans. To overcome this limitation, further clinical proof-of-concept studies that target GSDMD and neutrophil generation for management of AMI heart failure are needed.

In summary, we found that genetic knockout or pharmacological inhibition of GSDMD significantly improved heart function after AMI. Furthermore, bone marrow transplantation from *Gsdmd*-knockout mice led to a similar improvement. We provide mechanistic insights into molecular regulation of inflammatory responses during an AMI; that is, bone marrow–derived and GSDMD-dependent neutrophil generation and mobilization contribute to the detrimental immunopathology after AMI. We anticipate that our studies may be broadly applicable to cardioprotective therapy, specifically targeting GSDMD and neutrophil production for improved ventricular remodeling and reduced heart failure after AMI.

## Methods

### Human studies

#### Study design.

The STEMI (ST-segment elevation myocardial infarction) follow-up registry was a prospective, longitudinal, multicenter registry study of patients hospitalized with first-time STEMI in east China (ChiCTR-IDR-16007765). All data on patient demographics, signs and symptoms, medication, clinical characteristics, and discharge information were collected from a clinically based registry.

#### Study population.

A total of 8 sites were included in our registry, with 3 academic hospitals in each geographic city and 5 tier 2 district-centered hospitals. In short, our registry covered both tier 3 and tier 2 hospitals with long-term follow-up data. From June 2016 to June 2018, first-time STEMI patients 18 years of age or older who survived at discharge were included. The only exclusion criterion was missing echo data within hospitalization. STEMI was defined as a chest pain lasting 30 minutes or longer together with an ST-segment elevation in 2 or more contiguous leads on a standard 12-lead electrocardiogram (≥2 mm in precordial leads and ≥1mm in the limb leads).

### Mice

Details for reagents and antibodies described in the Methods can be found in Supplemental Table 2.

Adult C57BL/6N mice were purchased from Shanghai SLAC Laboratory. C57BL/6N *Gsdmd*^−/−^ mice were purchased from GemPharmatech. All animals were housed in a pathogen-free environment in the Tongji University animal facilities. Knockout of the *Gsdmd* gene was validated by genotyping and immunoblotting. All animal surgeries were performed in 10- to 14-week-old male mice. 

To deplete neutrophils in mice, isotype IgG (Cell Signaling Technology), anti-Ly6G antibody (BioLegend), or anti-Ly6G/Ly6C antibody (BioLegend) was injected intraperitoneally at a dose of 200 μg per mouse, as described previously (30, 31). The relevant mice were subjected to myocardial infarction surgery 24 hours later. For NSA administration, 20 mg/kg mouse weight of NSA (MedChemExpress) was injected intraperitoneally 30 minutes before AMI surgery; a second injection was administered 8 hours after the surgery. Mice in the corresponding control groups were injected with the same amount of solvent (DMSO) without NSA.

### Myocardial infarction

Mice were intubated and ventilated with 1% to 2% isoflurane. After left thoracotomy, the left coronary artery was ligated with a 6-0 polyester suture 1 mm below the left atrial appendage. Core temperature was monitored and maintained at 37°C, and the ECG was monitored to document ST-segment elevation during coronary occlusion. Surgeries were performed blinded to the genotypes. At corresponding time points, the mice were euthanized and the samples harvested.

### Myocardial ischemia reperfusion and 2,3,5-triphenyltetrazolium chloride staining

Mice were anesthetized by spontaneous inhalation of isoflurane and maintained under general anesthesia with 1% isoflurane. A left coronary artery occlusion was performed; 30 minutes later, the occlusion was reperfused for 24 hours prior to euthanization. The hearts were then harvested, dissected, and stained with Evans blue dye and 2,3,5-triphenyltetrazolium chloride. The images were captured under a Leica microscope and the ischemic area at risk and the area of necrosis were quantified. Quantification of the infarct area was normalized as a percentage of the nonperfused risk area during coronary occlusion.

### Echocardiography

Echocardiography was performed before the AMI and 7 days after AMI using a Vevo 2100 system (VisualSonics). Mice were kept under light anesthesia, and ultrasound gel was placed in the shaved chest and the probe was adjusted to a stable position. Midventricular M-mode echocardiograms were acquired at the level of the papillary muscles. Heart rate, intraventricular septum and posterior wall thickness, and end-diastolic and end-systolic internal dimensions of the left ventricle were obtained from the M-mode image.

### Protein analysis by immunoblotting or ELISA

For heart tissue samples, hearts were harvested from mice, rinsed with cold PBS, and divided into left and right ventricles. The tissues were immediately frozen in liquid nitrogen and then transferred to –80°C before homogenization. Total protein from tissues or cells was extracted in RIPA buffer (Cell Signaling Technology) supplemented with protease and phosphatase inhibitor cocktail (Roche). Total protein concentration was determined by BCA assay (Pierce). Protein was denatured by mixing with LDS sample buffer (GenScript) and β-ME (Amresco) and heating. Equal amounts of protein were loaded in SurePAGE gels (GenScript) and subjected to gel electrophoresis, followed by blotting onto PVDF membranes (Millipore). Western blotting analysis was performed using antibodies against GSDMD (Abcam), NLRP3 (Cell Signaling Technology), caspase-1 (Adipogen), IL-1β (R&D Systems), HSP90 (Cell Signaling Technology), and β-tubulin (Cell Signaling Technology), followed by HRP-conjugated secondary antibodies (Invitrogen). The blot was visualized with SuperSignal West Femto substrate (Pierce) on a ChemiDoc Imaging System (Bio-Rad) and analyzed in ImageJ (NIH). To test secretion levels of IL-1β and MCP-1 from leukocytes in the infarcted heart, hearts were harvested, rinsed in cold PBS, minced into small pieces, and single-cell suspensions were prepared and myeloid-originated cells were isolated using an anti-CD11b antibody and a cell isolation kit with LS Column (Miltenyi Biotec) according to the manufacturer’s instructions. The isolated myeloid-originated cells were then counted and cell numbers were adjusted to a same level, the cells were cultured for 24 hours, and the supernatants were harvested, purified by centrifugation, and further tested in ELISAs. The cell supernatant was tested for IL-1β levels using a Mouse IL-1 beta/IL-1F2 Quantikine ELISA Kit (R&D Systems) and LDH levels with a CytoTox 96 Non-Radioactive Cytotoxicity Assay (Promega) according to the manufacturers’ instructions. To test IL-1β or LDH levels in the serum, blood was drawn from the apex of the heart, rested in a microcentrifuge tube for 30 minutes, and centrifuged at 1,500*g* for 15 minutes; the serum supernatant was aliquoted and stored at –80°C until experiments.

### Bone marrow transplantation

The recipient mice were given acidic water (pH 2.6) supplemented with neomycin and polymyxin B (both from BBI Life Sciences Corp) 1 week prior to irradiation. The irradiation was administered using a small-animal X-ray irradiator (Rad Source) at a dose of 8 Gy. The donor bone marrow cells were isolated from the femurs of donor mice and injected into the recipient mice (5 × 10^6^ cells per mouse) by tail vein injection within 4 hours after the irradiation. The recipient mice were housed for 4 more weeks prior to myocardial infarction surgery. Spleen tissues were harvested and immunoblotting was performed to confirm that the bone marrow cells had been replaced.

### Immunofluorescence, TUNEL, and Masson’s trichrome staining

The hearts were harvested from euthanized mice, perfused from the apex with cold PBS to remove contaminating blood, embedded in OCT (Sakura), and then flash frozen. Frozen sections (5 μm) from mouse hearts were prepared for immunofluorescence staining. The sections were fixed with 4% paraformaldehyde for 30 minutes and washed in PBS, and antigen retrieval was performed by boiling the sections in sodium citrate. The sections were then permeabilized in 1% Triton X-100 (Sigma-Aldrich) for 10 minutes and blocked in 1% BSA for 30 minutes. Primary antibodies were diluted in 1% BSA and the sections were incubated with diluted antibodies at 4°C overnight. The sections were then washed in PBS, incubated with diluted secondary antibodies, and washed in PBS again. Subsequently, the sections were stained with DAPI (Invitrogen) and mounted with Prolong Gold Antifade Mountant (Invitrogen). The slides were scanned using a Nikon confocal microscope and quantification was performed in ImageJ. The immunofluorescence staining was performed using antibodies against CD68 (Abcam), MPO (R&D Systems), and α-actinin (Abcam). For TUNEL staining, 5-μm frozen sections were stained using an In Situ Cell Death Detection Kit (Roche) according to the manufacturer’s instructions. For Masson’s trichrome staining, 10-μm frozen sections from mouse hearts were prepared and the sections were stained using a Masson’s Trichrome Kit (Nanjing Jiancheng Bioengineering Institute) according to the manufacturer’s instructions. The images were captured using a Leica microscope and the images of 7 sections were used for the quantification of fibrotic area and ventricular wall thickness.

### Flow cytometry

Leukocytes from blood, bone marrow, and heart were analyzed by flow cytometry. Blood was drawn directly from the apex into centrifuge tubes containing 3.8% sodium citrate and the red blood cells were lysed with RBC lysis buffer (BioLegend). The cells were centrifuged at 400*g* and 4°C for 3 minutes and the cell pellets were resuspended in diluted antibodies. To isolate leukocytes from bone marrow, femurs from mice were isolated, cleaned, and the ends were cut open using scissors. The bone marrow was flushed with cold PBS using a 23-gauge needle, filtered through a 100-μm strainer, and centrifuged at 400*g* and 4°C for 3 minutes. The supernatant was discarded, the red blood cells were removed using RBC lysis buffer, and the cells were washed before resuspension in diluted antibodies. To analyze leukocytes in the heart, single-cell suspensions were initially prepared. Hearts were harvested, rinsed with cold PBS, and minced into pieces. The tissues were incubated in a cocktail of collagenase I (450 U/mL), collagenase XI (125 U/mL), hyaluronidase type I-s (60 U/mL), and DNase (60 U/mL) (all from Sigma-Aldrich) at 37°C for 1 hour with gentle agitation. After digestion, the single-cell suspensions were then filtered through a 100-μm strainer into a 50 mL tube, rinsed with FACS buffer, and centrifuged at 400*g* for 3 minutes. The cell pellets were resuspended in diluted antibodies and incubated at room temperature for 30 minutes in the dark. Antibodies against the following proteins were used: CD45-BV605, CD11b–Alexa Fluor 647, Ly6G-FITC, and Ly6C–Perp-Cy5.5 (all from BioLegend). Data were acquired on an LSRFortessa flow cytometer (BD Biosciences) and analyzed in FlowJo (version 10.6.2). The myeloid leukocytes were identified as CD45^+^CD11b^+^ and further classified as Ly6G^+^ neutrophils or Ly6C^+^ monocytes.

### Myeloid-derived cell and neutrophil isolation and culture

In order to explore the function of myeloid-derived cells and neutrophils, we isolated neutrophils in the heart by using the CD11b MicroBeads UltraPure or the Neutrophil Separation kit (Miltenyi Biotec) according to the manufacturer’s instructions, as described previously (32). Myeloid-derived cells and neutrophils were isolated from the heart of mice at different time points after myocardial infarction, followed by immunoblotting or ELISA. The viability of the neutrophils cultured for 24 and 72 hours was determined with an annexin V/PI staining kit (Thermo Fisher Scientific), followed by flow cytometry as described previously (33, 34).

### RNA-Seq data processing

Total RNAs derived from whole hearts 1 and 7 days after AMI and sham were applied for whole-transcriptome sequencing. Briefly, a cDNA library was prepared using random hexamer primers and PCR amplification. PCR products were purified (AMPure XP system) and library quality was assessed on the Agilent Bioanalyzer 2100 system. After cluster generation via using TruSeq PE Cluster Kit v3-cBot-HS (Illumina), the library preparations were sequenced on an Illumina Hiseq platform and 150-bp paired-end reads were generated.

The quality of the reads was evaluated with FastQC (https://www.bioinformatics.babraham.ac.uk/projects/fastqc/). The reads were then trimmed with Cutadapt (https://github.com/marcelm/cutadapt/) to remove low quality bases and remove adapters. Alignment of the resulting high-quality reads to the mouse reference Ensembl version GRCm38.92 (https://asia.ensembl.org/) was performed via the splice-aware aligner STAR v2.4.0j (https://github.com/alexdobin/STAR). Afterwards, using RNA-Seq by expectation maximization (RSEM; https://github.com/deweylab/RSEM), the abundance of each gene was quantified as transcripts per million (TPM). All original data were deposited in the NCBI’s Gene Expression Omnibus database (GEO GSE181872).

### Principal component analysis

We calculated the standard deviation (SD) of each gene across samples and selected those with an SD of 0.5 or greater to generate first and second principal components with the unsupervised learning technique, Principal Component Analysis (PCA).

### Analysis of DEGs

The DEGs, defined by fold change (FC) of 2 or greater and a false discovery rate (FDR) of less than 0.05, were called using DESeq2 (https://github.com/mikelove/DESeq2) and were used to generate a hierarchical clustering with the “pheatmap” package in R (v4.0.4, https://www.r-project.org/). The intersections of DEGs created from different comparisons were calculated via the “VennDiagram” package. DEGs that had coherent expression patterns across sham, AMI (day 1), and AMI (day 7) were further clustered together. The genes in different patterns were respectively mapped onto the GO, and the adjusted *P* value indicating whether a function was enriched by DEGs was calculated using the hypergeometric distribution.

### Statistics

All data are presented as mean ± SD. Comparison of 2 groups was performed by an unpaired, 2-tailed Student’s *t* test. When more than 2 groups were compared, statistical significance was determined using 1-way ANOVA followed by Tukey’s multiple comparison test or Bonferroni’s multiple comparison test. A *P* value of less than 0.05 was considered to indicate significance. Statistical significance of Kaplan-Meier survival curves was determined by the Mantel-Cox test. Relationships between variables were determined by Pearson’s correlation coefficient. Statistical analyses were performed with Prism (GraphPad Software, version 8.3.0).

### Study approval

For animal studies, all animal experiments were approved by the Tongji University Animal Research Committee (TJLAL-019-128). Human studies followed the principles of the Declaration of Helsinki and were approved centrally by the Ethics Committee at Shanghai Chest Hospital, Shanghai Jiao Tong University (approved number 2015-111), and by the local health research ethics board at each participating hospital. Written informed consent was obtained from each patient to allow for follow-up data.

## Author contributions

Y Xiang designed the study. KJ, ZT, KC, FC, SX, TS, and DW performed the animal experiments and the in vitro experiments. Y Xiang, KC, KJ, Y Xu, JQ, LS, and JH analyzed the data. Y Xiang wrote the manuscript. KJ, ZT, and KC are co–first authors based on their distinct contributions; the order of co–first authors was determined based on the overall scientific contribution.

## Supplementary Material

Supplemental data

## Version 1 11/09/2021

In-Press Preview

## Version 2 01/04/2022

Electronic publication
