# Supplementary material for: Gasdermin D inhibition confers antineutrophil-mediated cardioprotection in acute myocardial infarction
Source: J Clin Invest. 2022 Jan 4;132(1):e151268. doi: 10.1172/JCI151268 (PMC8718151; doi:10.1172/JCI151268)
Supplement: Supplemental data [file jci-132-151268-s009.pdf]

Supplemental Figure 1

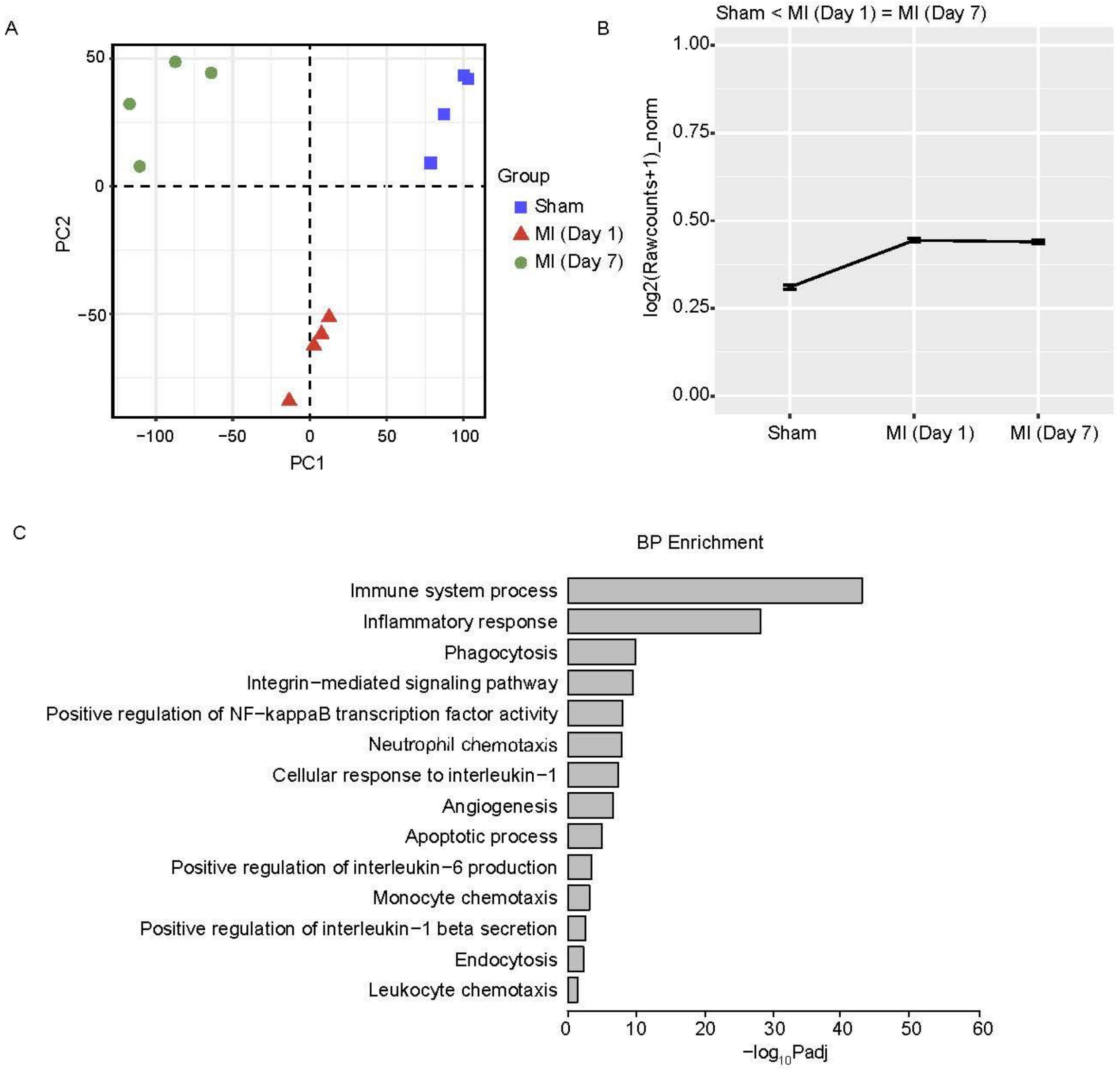

Figure S1. Gene expression analysis by RNA-Seq.

(A) The distribution of samples was determined by the 1st and 2nd principal component generated by principal component analysis (PCA). **(B)** Represented one of the dynamic expression patterns of genes across Sham, MI (Day 1) and MI (Day 7), with **(C)** showing the functional analysis of such genes by GO Biological Process based on hypergeometric distribution (the adjusted P-value < 0.05).

Supplemental Figure 2

A

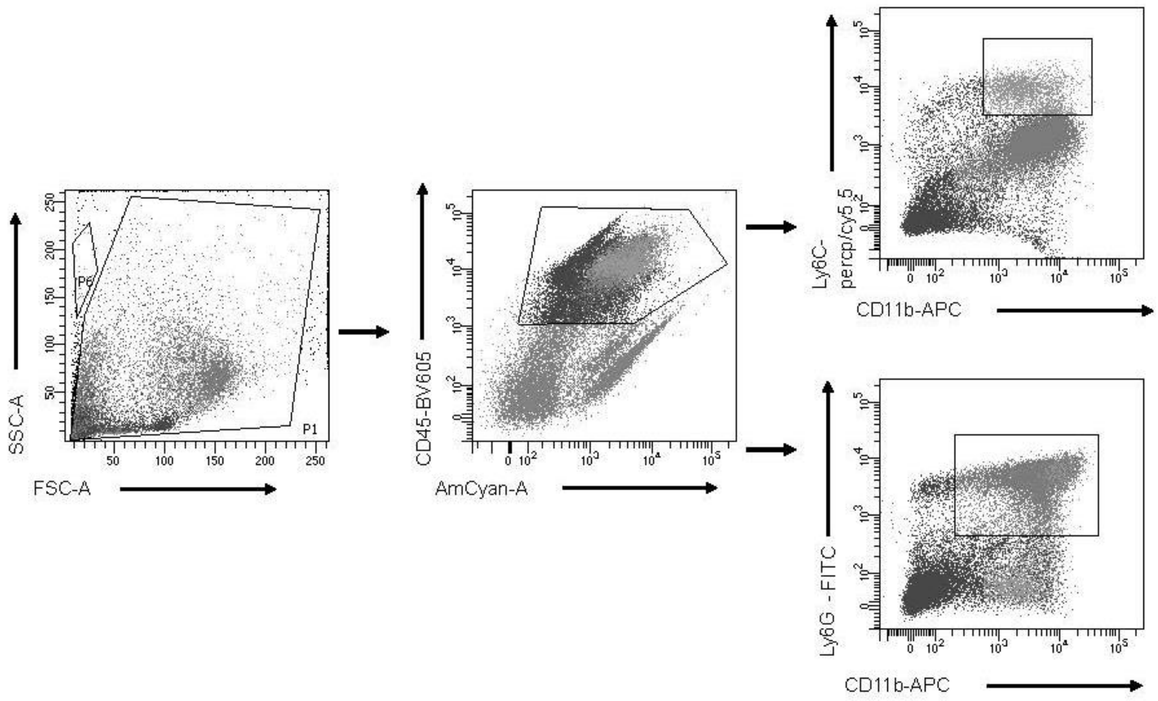

Figure S2. Gating strategy for flow cytometric analysis.

Supplemental Figure 3

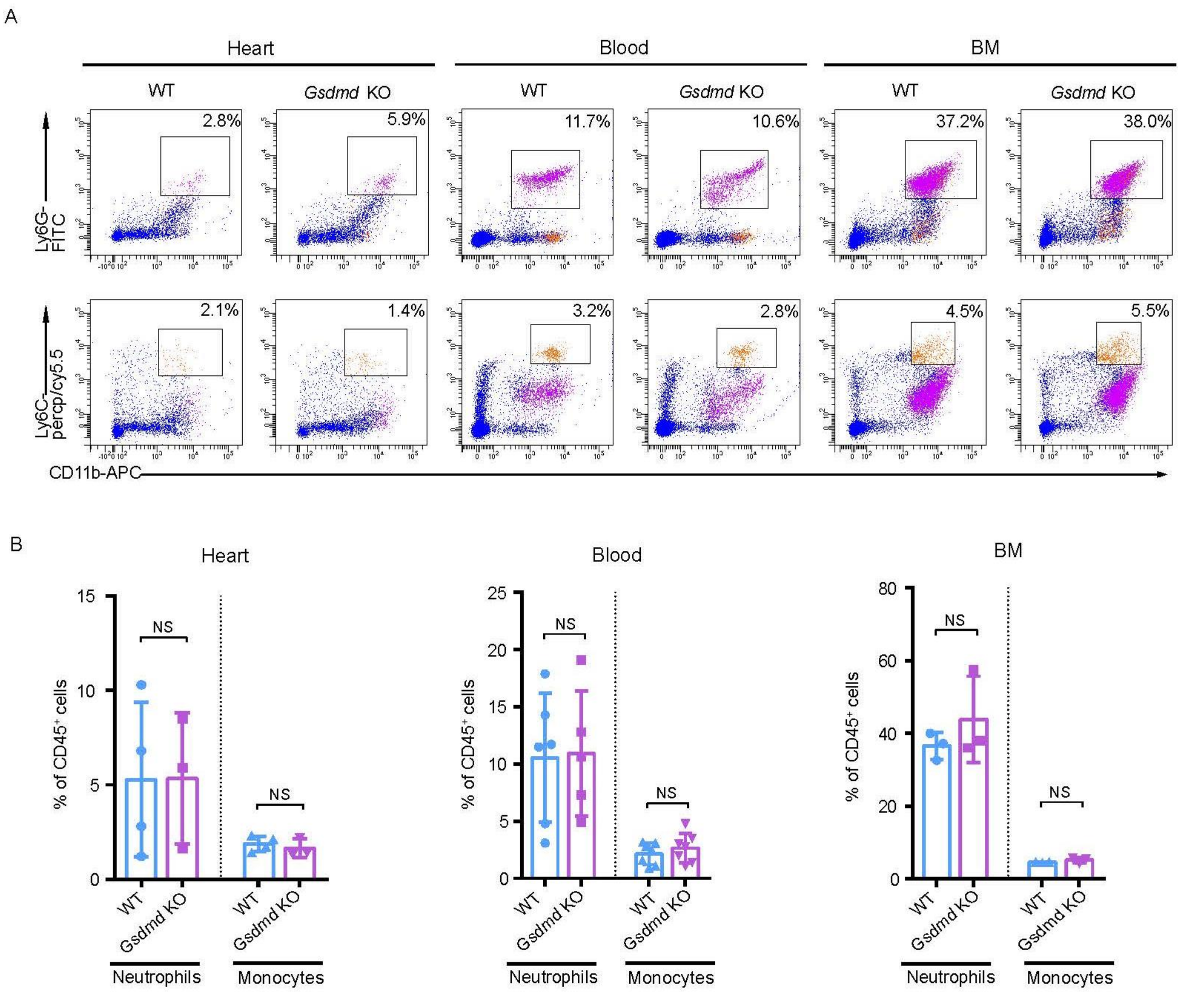

Figure S3. The baseline of WT or *Gsdmd*$^{-/-}$ mice leukocytes.

Flow cytometric analysis **(A)** and quantification **(B)** of Cd11b^{+}Ly6G^{+} neutrophils and Cd11b^{+}Ly6C^{+} monocytes in blood from WT or *Gsdmd*$^{-/-}$ mice at baseline without a MI surgery (n= 3-7). Data are mean ± SD and were analyzed by unpaired two-tailed Student's *t* test **(B)**. NS, not significant.

Supplemental Figure 4

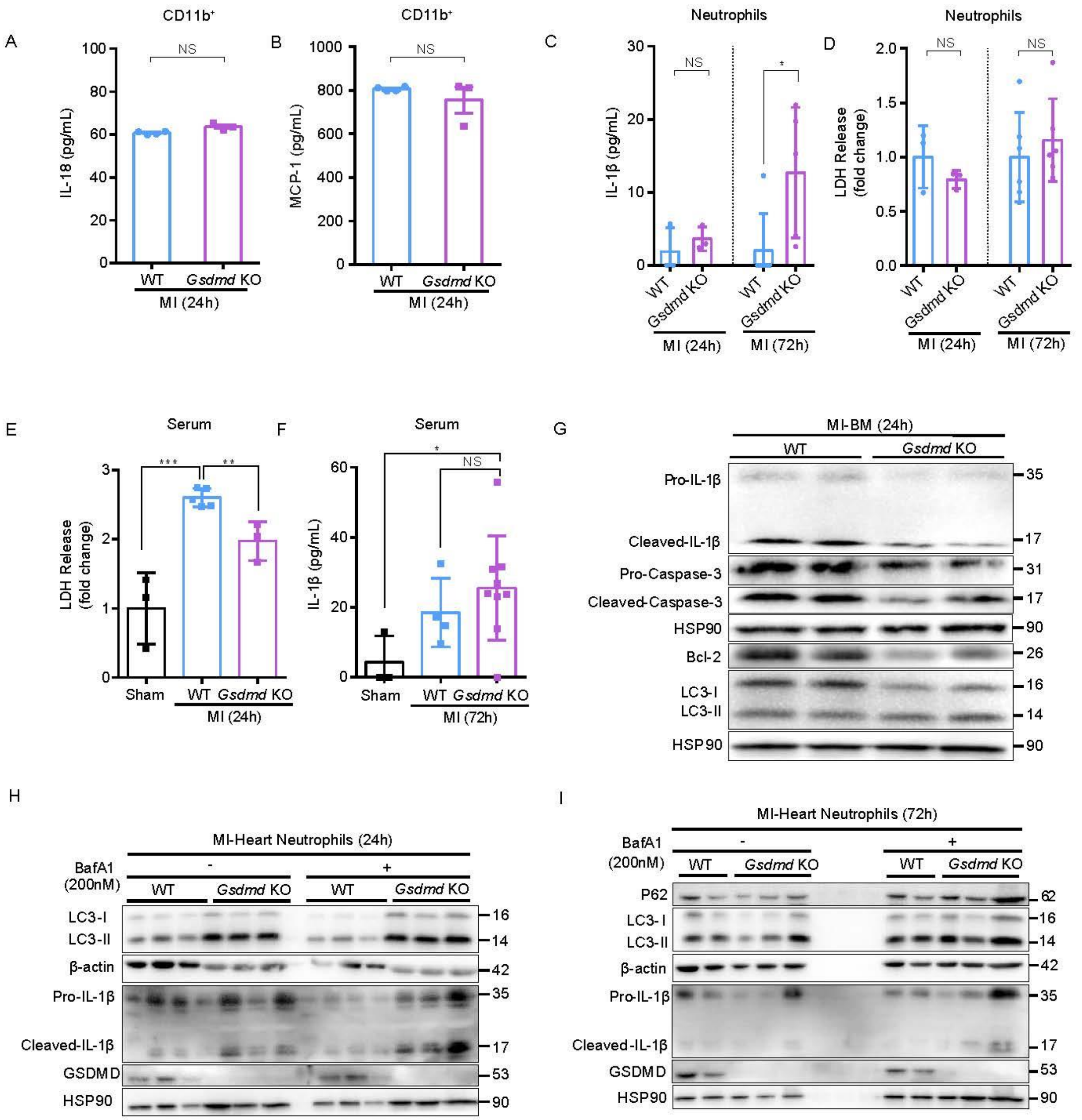

Figure S4. Biochemical analysis of myeloid-originated cells from the heart.

(A-B) Secretion levels of IL-18 and MCP-1 from myeloid-originated cells from the heart of WT (n = 4) or *Gsdmd*$^{-/-}$ (n = 3) mice at 24 hours after MI assessed by ELISA. **(C)** Secretion levels of LDH from neutrophils (2 ×10^{6}) from the heart of WT or *Gsdmd*$^{-/-}$ mice at 24 hours and 72 hours after MI. **(D)** Production of IL-1β from neutrophils from the heart of WT or *Gsdmd*$^{-/-}$ mice at 24 hours and

72 hours after AMI assessed by ELISA. The corresponding n values were indicated in the plot. **(E)** Serum levels of LDH from WT or *Gsdmd*$^{-/-}$ mice at 24 hours after MI or a sham surgery. **(F)** Serum levels of IL-1β from WT or *Gsdmd*$^{-/-}$ mice at 72 hours after MI or a sham surgery. **(G)** Representative immunoblotting images of protein levels of BM cells from WT or *Gsdmd*$^{-/-}$ mice at 24 hours after MI or a sham surgery (n = 2 per group). **(H-I)** Representative immunoblotting images of protein levels of neutrophils cells with or without BafA1 (200 nM) from the heart of WT or *Gsdmd*$^{-/-}$ mice at 24 hours or 72 hours after MI (n = 2 - 3 per group). Data represent mean ± SD and were analyzed by unpaired two-tailed Student's *t* test **(A, C and D)** or One-way analysis of variance followed by Tukey's multiple comparison test **(E)**. NS, not significant; *, $P<0.05$; **, $P<0.01$; ***, $P<0.001$.

Supplemental Figure 5

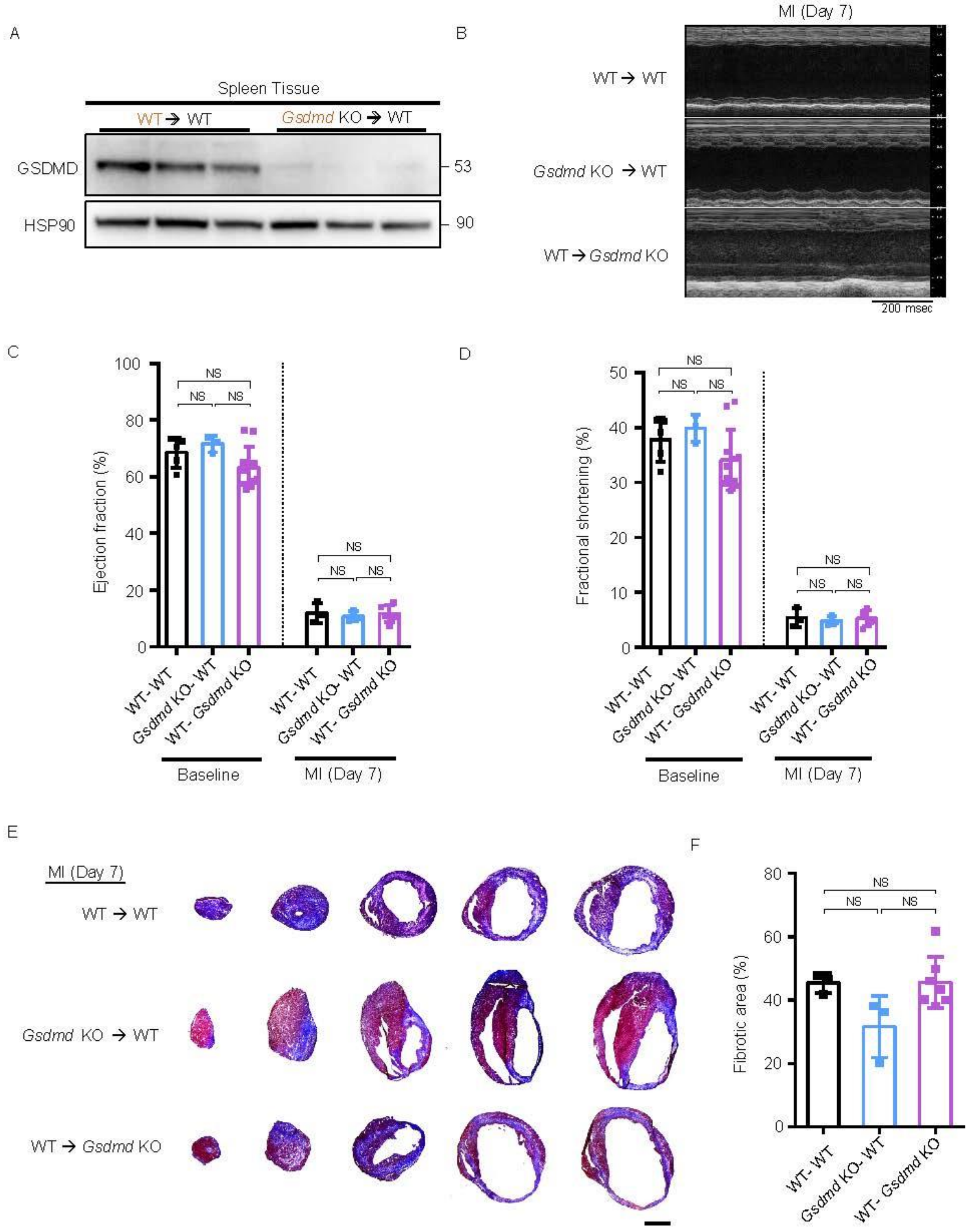

41

Figure S5. Bone marrow transplantation and functional analysis of mice.

(A) Immunoblotting for GSDMD validating the success of BMT experiment. **(B-D)** Echocardiography images **(B)** and M-mode quantification of ejection fraction **(C)** and fractional shortening **(D)** for WT or *Gsdmd*$^{-/-}$ mice before or at 1 week after MI (baseline: WT → WT, n = 5; *Gsdmd* KO → WT, n = 3; WT → *Gsdmd* KO, n=11; 1 week: WT → WT, n = 3; *Gsdmd* KO→WT, n = 3; WT→ *Gsdmd* KO, n=5). **(E-F)** Masson's Trichrome staining **(E)** and quantification **(F)** of fibrotic area of short-axis heart sections from WT → WT (n = 3), *Gsdmd* KO → WT (n = 3) or WT→ *Gsdmd* KO (n=7) mice at 1 week after MI (scale bar, 1 mm). Data are mean ± SD and were analyzed by One-way analysis of variance followed by Tukey’s multiple comparison test **(C, D and F)**. NS, not significant.

Supplementary Figure 6

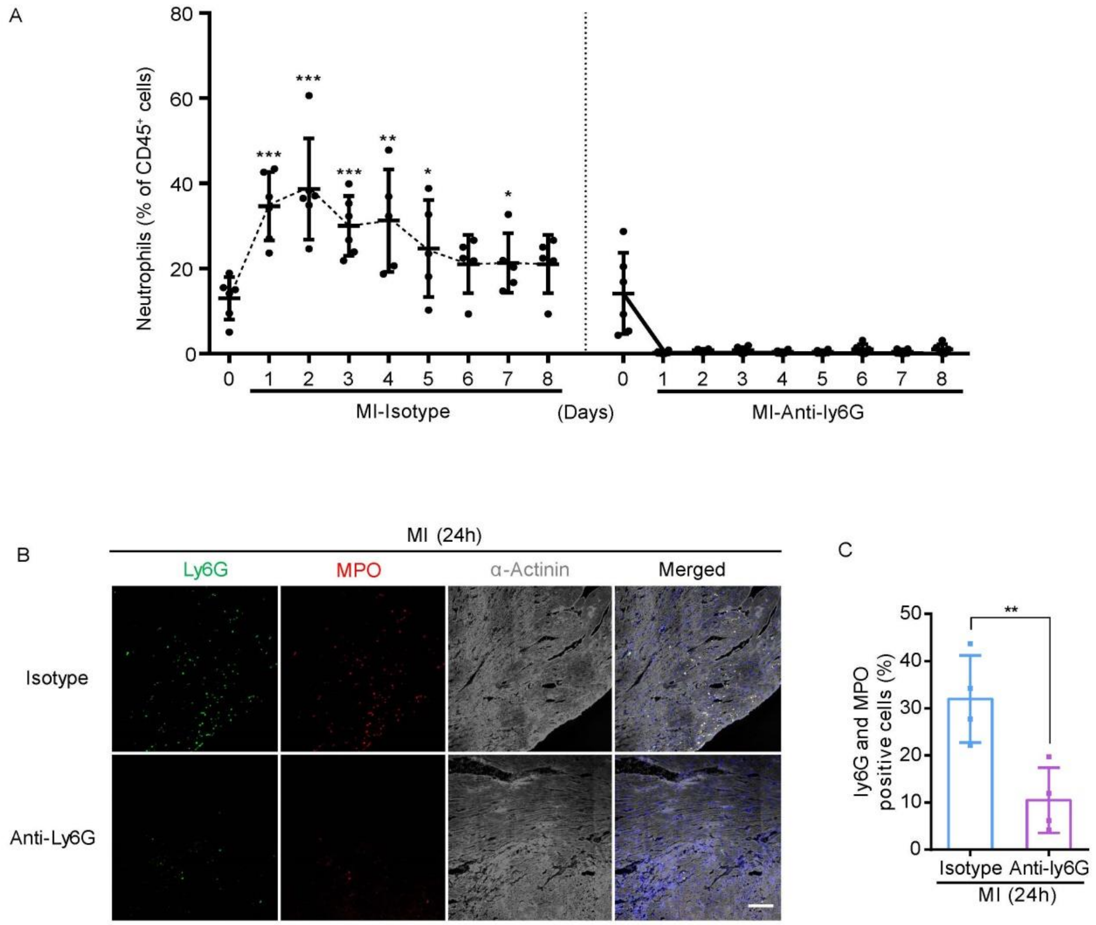

Figure S6. Validation of Cd11b$^+$Ly6G neutrophils depletion

(A) Flow cytometric quantification of Cd11b$^+$Ly6G$^+$ neutrophils in blood from WT or anti-ly6G treated mice at different time points. The corresponding n values were indicated in the plot. **(B)** Immunofluorescence imaging on heart sections from Isotype or Anti-ly6G mice at 24h after MI showing α-Actinin (gray), Ly6G (green), MPO (red) and DAPI (blue). Representative fields are presented (scale bar, 100 μm). **(C)** Quantification of Ly6G and MPO positive area proportion in the field of heart sections from Isotype or Anti-ly6G mice, each value was averaged from the values of 5 fields of view from the same mouse (n = 4 per group). Data represent mean ± SD and

were analyzed by One-way analysis of variance followed by Tukey’s multiple comparison test **(A)** or unpaired two-tailed Student’s *t* test **(C)**. NS, not significant; *, P<0.05; **, P<0.01; ***, P<0.001; ****, P<0.0001.

Supplementary Figure 7

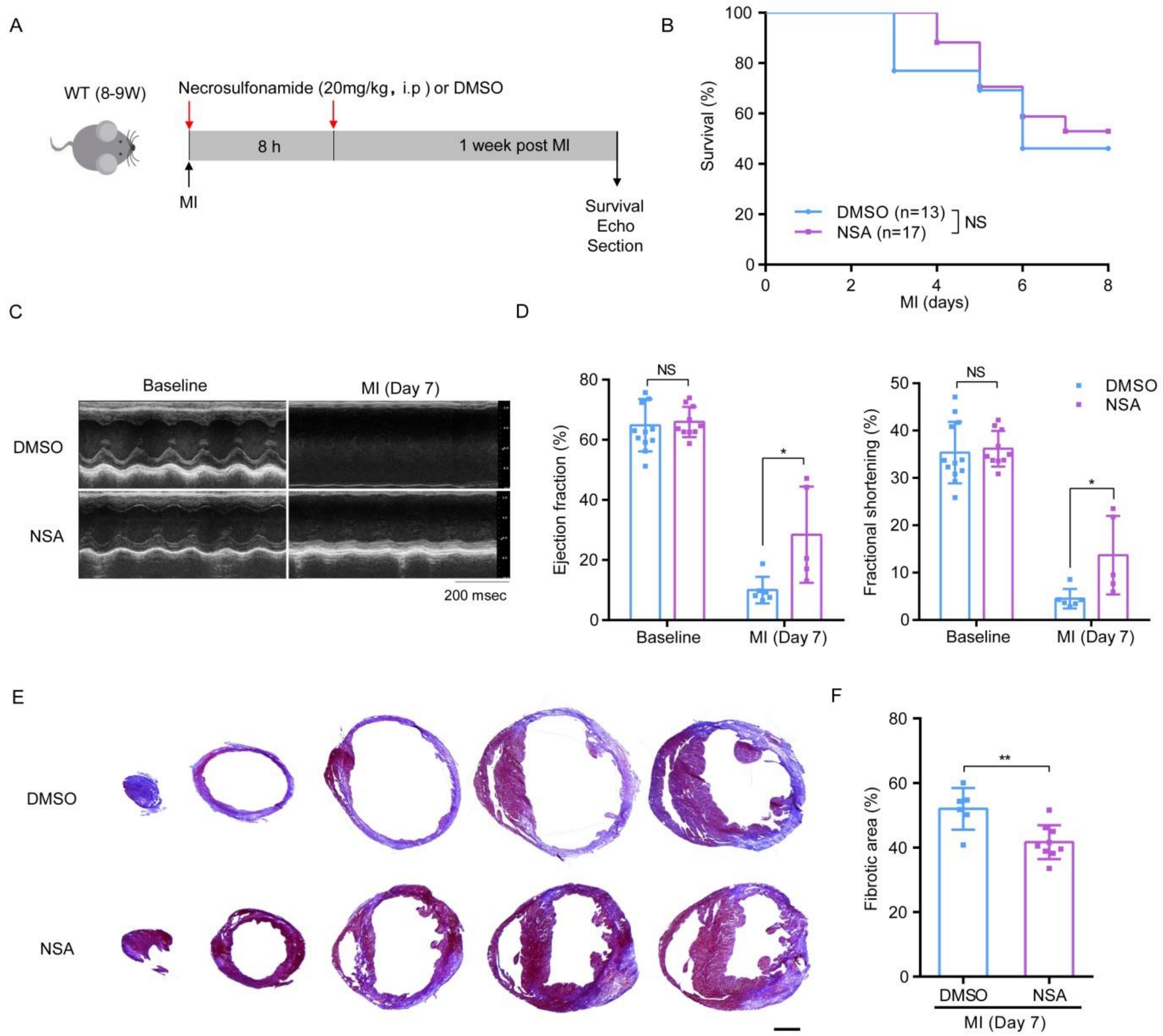

Figure S7. Pharmacological inhibition of GSDMD reduces infarct size post MI.

(A) Schematic diagram showing the strategy of postoperative NSA administration to the mice. **(B)** Kaplan-Meier survival curves comparing post-MI survival of control (DMSO administration) mice (n = 13) to that of mice administrated with NSA (n = 17). Significance was determined by Mantel-Cox test. **(C-D)** Echocardiography images **(C)** and M-mode quantification **(D)** of ejection fraction (left) and fractional shortening (right) for control mice or mice with NSA administration before or at 1 week after MI. (baseline: DMSO, n = 12; NSA, = 10; 1 week: DMSO, n = 6; NSA, n

= 5). **(E-F)** Masson's Trichrome staining **(E)** and quantification of fibrotic area **(F)** of short-axis heart sections from control mice or mice with NSA administration at 1 week after MI (DMSO, n = 6; NSA, n = 9) (scale bar, 1 mm). Data are mean ± SD and were analyzed by One-way analysis of variance followed by Tukey’s multiple comparison test **(D)** or unpaired two-tailed Student’s *t* test **(F)**. NS, not significant; *, P<0.05; **, *P*<0.01.

Supplemental Table 1: Patients' characteristics.

**Demographics**	**n=234**
Age (years)	**63.97±11.991**
Sex (Male/Female)	**41/193**
**Clinical history**	
Previous PCI, n(%)	**5(2.1)**
Previous CABG, n(%)	**0(0)**
Previous Stroke, n(%)	**13(5.6)**
Hypertension, n(%)	**111(47.4)**
Diabetes, n(%)	**41(17.5)**
Dyslipidemia, n(%)	**2(0.9)**
**Medications**	
β-blockers, n(%)	**176(75.2)**
Aspirin, n(%)	**216(92.3)**
Statin, n(%)	**215(91.9)**
ACEI/ARB, n(%)	**149(63.7)**
ADP inhibitors, n(%)	**217(92.7)**

Supplemental Table 2: Key Resources and Reagents

REAGENT or RESOURCE	SOURCE	IDENTIFIER
Anti-GSDMD	Abcam	Ab209845
Anti-CD68	Abcam	Ab53444
Anti-Sarcomeric Alpha Actinin	Abcam	Ab9465
Caspase-1+p10+p20	Abcam	Ab179515
Anti-mouse Caspase-1	Adipogen	AG-20B-0042-C100
Anti-mouse IL-1β	R&D Systems	AF-401-NA
MPO	R&D Systems	AF3667
Bcl-2	SantaCruz	Sc-7382
Anti-mouse NLRP3	Cell Signaling	1510S
SQSTM1/P62 Antibody	Cell Signaling	5114S
LC3 A/B Antibody	Cell Signaling	4108S
Caspase-3	Cell Signaling	9662S
HSP90	Cell Signaling	4877S
β-actin	Cell Signaling	4970S
β-tubulin	Cell Signaling	2146S
GAPDH	Cell Signaling	3683S
anti-Ly6G/Ly6C antibody	BioLegend	108436
CD45-BV605	BioLegend	103140
CD11b-AlexaFluor647	BioLegend	101218
Ly6G-FITC	BioLegend	127606
Ly6C-Perp-cy5.5	BioLegend	128012
Anti-Ly6G antibody	BioLegend	127632
Goat anti-mouse IgG, HRP	Invitrogen	31430
Goat anti-rabbit IgG, HRP	Invitrogen	31460
TEXAS-Red goat anti-rat IgG	Invitrogen	T2767
Alexa Fluor 488 goat anti-rat IgG	Invitrogen	A11034
Alexa Fluor 594 goat anti-mouse IgG	Invitrogen	A11032
Alexa Fluor 488 goat anti-rat IgG	Invitrogen	A11006
Alexa Fluor 555 donkey anti-goat IgG	Invitrogen	A21432
Alexa Fluor 488 goat anti-mouse IgG	Invitrogen	A11001
Alexa Fluor 647 goat anti-rat IgG	Invitrogen	A21247
CD11b microbeads UltraPure mouse	Miltenyi	130-126-725
Neutrophil separation kit	Miltenyi	130-097-658
Mouse chow	Changzhou SYSE Bio-Tec.Co.Ltd	PD450J
Bafilomycin A1	MCE	HY-100558
